# Hedgehog Signaling: Implications in Cancers and Viral Infections

**DOI:** 10.3390/ijms22031042

**Published:** 2021-01-21

**Authors:** Sidney Iriana, Kumari Asha, Miroslava Repak, Neelam Sharma-Walia

**Affiliations:** Center for Cancer Cell Biology, Immunology and Infection, Department of Microbiology and Immunology, Chicago Medical School, Rosalind Franklin University of Medicine and Science, North Chicago, IL 60064, USA; Sidney.iriana@my.rfums.org (S.I.); asha.kumari@rosalindfranklin.edu (K.A.); miroslava.repak@my.rfums.org (M.R.)

**Keywords:** GLI transcription factors, cancer, virus, therapeutic

## Abstract

The hedgehog (SHH) signaling pathway is primarily involved in embryonic gut development, smooth muscle differentiation, cell proliferation, adult tissue homeostasis, tissue repair following injury, and tissue polarity during the development of vertebrate and invertebrate organisms. GLIoma-associated oncogene homolog (GLI) family of zinc-finger transcription factors and smoothened (SMO) are the signal transducers of the SHH pathway. Both SHH ligand-dependent and independent mechanisms activate GLI proteins. Various transcriptional mechanisms, posttranslational modifications (phosphorylation, ubiquitination, proteolytic processing, SUMOylation, and acetylation), and nuclear-cytoplasmic shuttling control the activity of SHH signaling pathway proteins. The dysregulated SHH pathway is associated with bone and soft tissue sarcomas, GLIomas, medulloblastomas, leukemias, and tumors of breast, lung, skin, prostate, brain, gastric, and pancreas. While extensively studied in development and sarcomas, GLI family proteins play an essential role in many host-pathogen interactions, including bacterial and viral infections and their associated cancers. Viruses hijack host GLI family transcription factors and their downstream signaling cascades to enhance the viral gene transcription required for replication and pathogenesis. In this review, we discuss a distinct role(s) of GLI proteins in the process of tumorigenesis and host-pathogen interactions in the context of viral infection-associated malignancies and cancers due to other causes. Here, we emphasize the potential of the Hedgehog (HH) pathway targeting as a potential anti-cancer therapeutic approach, which in the future could also be tested in infection-associated fatalities.

## 1. Introduction

Hedgehog (HH) signaling is a necessary, evolutionarily conserved developmental process for human embryogenesis and organogenesis. First identified in *Drosophila melanogaster*, HH signaling is extensively used to explain the complex topic of cell segment number patterning. Advanced research identified its important morphogenic properties for cell proliferation, polarity, and differentiation [1]. Mammals have three HH homologs, Desert (DHH), Indian (IHH), and Sonic (SHH), of which Sonic is the best-studied ligand of the vertebrate pathway [1]. SHH signaling promotes adult stem cells’ proliferation, including primitive hematopoietic [2], mammary, and neural stem cells [3]. Oncogenic mechanisms, however, can alter their fates in adult tissues and cause aberrant upregulation of the HH signaling pathway. Oncogenic mechanisms include cell proliferative capacity, angiogenesis, epithelial to mesenchymal transition (EMT), and invasive migration patterns typical of metastatic tissues [4,5,6]. With its first discovery in Gorlin syndrome, also known as nevoid basal cell carcinoma syndrome [7,8], HH signaling has been explored in gastrointestinal (GI), ovarian, brain, lung, skin, prostate, and breast cancers [9]. BCC and medulloblastoma tumors have constitutive activation of the HH pathway [10]. Downstream HH signaling protein families such as GLI have become the spotlight as targets for invasive epithelial cancers. HH pathway has been investigated for potential therapeutic antagonists as a strategy for cancer treatment, although the focus is limited to ligand-dependent effectors such as Patched 1 (PTCH1) and Smoothened (SMO); Frizzled class receptor [11,12]. In this review, we discuss the prospect of HH/GLI signaling in cancers due to viral infections and other causes.

## 2. Various Types of GLI

**Figure 1:**
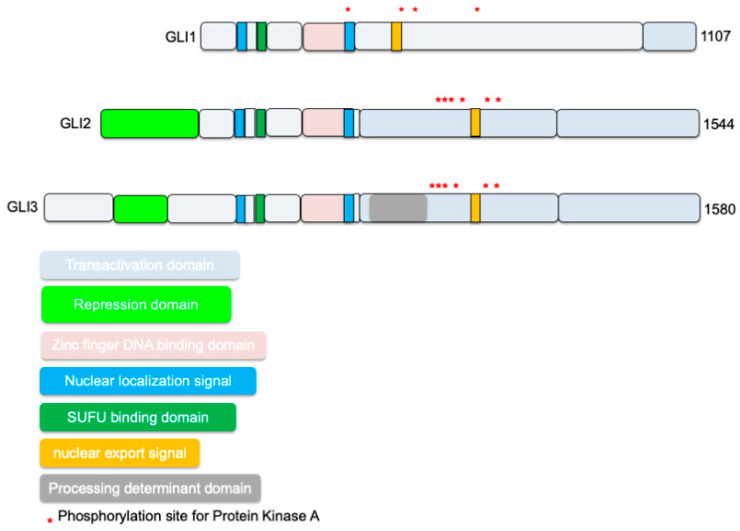
Schematic shows known full-length GLIoma-associated oncogene homolog (GLI) family functional domains at the transcriptional level. Similarities shared among all three types include Suppressor of fused (SUFU) binding sites, zinc finger DNA-binding domain, and activation domains. GLI1 has an additional SUFU binding site at the C-terminus, while GLI2/3 has repressor domains; additionally, GLI2 has an extra activation domain that supports its main feature of activating GLI-mediated transcription.

The GLI family includes three zinc finger proteins GLI1, GLI2, and GLI3 transcription factors identified as downstream targets of the HH signaling pathway—important in proper embryonic development, stem cell biology, and tissue homeostasis. Of the GLI proteins, GLI2 and GLI3 are the primary signal responders, with GLI2 mainly acting as a transcriptional activator and GLI3 predominantly acting as a repressor [13] (Figure 1). GLI1, a direct transcriptional target of HH signaling, is dispensable for mouse embryonic development [14,15]. HH signaling is GLI1 dependent in the spinal cord, and a combination of GLI2 and GLI3 is required to regulate motor neuron and early ventral spinal cord development [16]. Structurally, they share similar features, including conserved tandem zinc fingers, conserved N- and C-terminal domains, and regions of protein kinase A (PKA) binding. There is an increased level of complexity in GLI transcription factors’ activation process as alternative splicing plays a significantly important role in controlling their activity. GLI1 alone functions as a transcriptional activator, whereas GLI2 and GLI3 have bifunctional transcription forms: full-length versions act as activators, and truncated N-terminal fragments act as repressors [13]. GLI2 and GLI3 display a repressor domain (RD), a processing determinant domain (PDD) [17], and a processing region (PR). N-terminal region of human GLI2 and GL13 contains a domain with a transcriptional repressor, and removal of this domain enhances the transcriptional activity of GLI2 [16,18,19]. The balance between the GLI2/3 transcriptional activator and repressor actions dictates HH responses that are both tissue-specific and developmental stage-specific. During limb and ureter development, SHH acts mainly by opposing GLI3 repression [13,20,21,22]. During neural tube and skeletal development, integrated regulation of GLI2 activation and GLI3 de-repression has both overlapping and distinct functions. Both GLI2 and GLI3 constitutively traffic through primary cilia until activation of the HH pathway promotes the parallel accumulation of GLI2 and GLI3 at the tip of primary cilia [23]. This region acts as an organizational center for activated GLI proteins, which then traffic to target gene promoters in the nucleus. The balance between these functional forms in the cell environment—which depend on post-transcriptional and post-translational processing—determines the HH transcription’s net direction and subsequent effects on tissue proliferation.

Figure 1 shows GLI family functional domains, their similarities at the transcriptional level. GLI proteins belong to the GLI-Kruppel family of transcription factors and have Kruppel-type zinc-finger (ZF) motifs in their DNA binding domains [24,25,26]. Suppressor of fused (SUFU) binding site is highly conserved across all three mammalian GLI proteins [24,25,26]. Localization of GLI1 is influenced by the presence of a nuclear export signal (NES), and GLI1 becomes constitutively nuclear when this signal is mutated, or nuclear export is inhibited [27]. SUFU is a conserved negative regulator of GLI1 signaling that may affect the nuclear-cytoplasmic shuttling of GLI1 or the activity of GLI1 in the nucleus and thereby modulate cellular responses [27]. Recently, a PY-type nuclear localization signal (PY-NLS) and the nuclear import factor karyopherin β2 (Kapβ2) were discovered to regulate GLI ciliary localization and HH pathway activity [28]. PY-NLS acts in conjunction with the canonical NLS (a bipartite NLS) localized in the Zn-finger domain [29]. The canonical nuclear localization signal (NLS) in GLI plays a significant role, whereas the proline-tyrosine or PY-NLS has a minor role in targeting GLI to the nucleus. Interestingly, mutating the PY-NLS but not the canonical NLS impaired GLI ciliary localization and PY-NLSs interact with the Kapβ2, also known as transportin 1 (TRN1) or importin β2 and transport PY-NLS-containing proteins to the nucleus [28]. GLI activity is restrained by the phosphorylation of six conserved serine residues by protein kinase A (PKA) in the absence of HH ligands [30]. GLI protein functions are regulated via post-translational modifications such as phosphorylation by PKA, glycogen synthase kinase 3 (GSK3), and casein kinase 1 (CK1) or ubiquitination, SUMOylation, acetylation, or methylation [24,25,26]. Two isoforms of GLI1 are termed GLI1ΔN and tGLI1 [31]. The full-length GLI1 is designated as GLI1FL [31]. However, GLI1ΔN lacks the SUFU-binding domain. It can activate and turn on target genes similarly to GLI1FL [19]. tGLI1 has a deletion of 41 amino acids, but it preserves all the functional domains present in GLI1FL. tGLI1 efficiently translocates into the nucleus to activate gene transcription and responds to HH ligand stimulation as well [6].

## 3. GLI and Hedgehog Signaling

**Figure 2:**
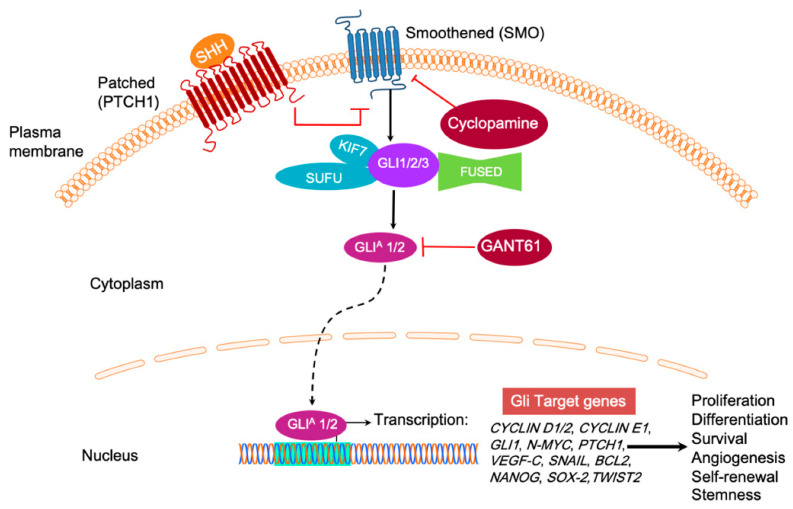
In the absence of Hedgehog (HH) ligand (SHH, IHH, and DHH), the Patched 1 (PTCH) transmembrane receptor at the base of the primary cilium maintains inhibition of Smoothened (SMO) G protein coupled receptor (GPCR) signaling. Upon HH binding, SMO inhibition is relieved and activates GLI transcription factors, usually sequestered by Suppressor of fused (SUFU), KIF7, and FUSED. GLIA refers to the transcriptionally active form of GLI. SMO is a popular drug target for cyclopamine, while GLI1 and GLI2-induced transcription can be inhibited by GANT61. It inhibits the HH signaling pathway downstream of SMO and SUFU, causing GLI1 nuclear accumulation. Target gene expression includes CYCLIN D1/2, CYCLIN E1, GLI1, N-MYC, Patched1, VEGF-C, and SNAIL to upregulate cell proliferation and tumor survival.

The classical Hedgehog signaling pathway depends on the secretion of extracellular HH glycoproteins, Sonic HH (SHH), Indian HH (IHH), or Desert HH (DHH) [1]. SHH plays a critical role in maintaining normal embryogenesis, and abnormal fetal concentrations throughout its vast expression territory leading to the developmental defects, while its absence is lethal [32]. IHH, produced in hematopoietic cells, bone, and cartilage, has prominent HH signaling roles in the fetal liver [33]. DHH resides mostly in the peripheral nervous system and the testes, where its expression in pre-Sertoli cells serves as a marker for male sexual differentiation [34]. Despite the macroscopic arrangement and responsibilities of the HH family, canonical signaling remains conserved to the interaction of activated HH ligand binding and inactivating the cell-surface transmembrane protein, PTCH1 (Figure 2) [35]. In the inactive HH pathway, PTCH1 blocks the migration of SMO, a G protein-coupled receptor (GPCR), to the cell membrane. When the SHH ligand binds to PTCH1, inhibition upon SMO, a neighboring GPCR, is subsequently relieved, triggering downstream activation and nuclear localization of transcription factors like those in the GLI family to regulate the expression of target HH genes, effectively inducing cell proliferation, survival, differentiation, and angiogenesis. In the absence of HH ligand binding, GLI proteins are typically sequestered in the cytoplasm by the negative regulator called SUFU, which directly binds GLI to prevent activation of downstream pathway genes (Figure 2) [36]. PKA initiates a phosphorylation cascade for PKA, GSK3, and CK1 phosphorylate GLI3 [37]. Phosphorylation of GLI3 targets it for ubiquitination [37]. Kinesin family member protein 7 (KIF7) is a conserved regulator of the HH signaling. KIF7 localizes to the cilium tip, the site of microtubule plus ends. KIF7 must relay the signals from the membrane protein SMO to the GLI family transcription factors [38]. KIF7 affects HH signaling, both positively and negatively (Figure 2). It functions as a negative regulator of the SHH pathway by preventing GLI2 activation in the absence of ligand, and as a positive regulator by preventing the processing of GLI3 into its repressor form. FUSED (FU), the putative serine/threonine kinase, does not function in the mammalian HH signaling but plays a role in motile cilia [39] (Figure 2). The balance between activated and repressed forms of the GLI transcription factor determines the fate of target gene expression and cell phenotype. 

## 4. GLI Code

GLI expression is tightly regulated at the transcription level by multiple signaling inputs—in the context of phase activity—that converge on the GLI code and direct its fate. Whether cells undergo healthy development, homeostasis, or have a dynamic expression of oncogenes and tumor suppressors during metastasis, GLI transcription responds appropriately to the cells’ desired phenotype’s instructional cues. For example, tumor suppressor p53 inhibits GLI1-induced neural stem cell self-renewal, proliferation, and tumor growth through repressing nuclear localization and transcription activity of GLI1 [40]. In contrast, the absence of p53, a hallmark event of most cancers, contributes to unregulated GLI1 expression and tumor progression [41]. Similarly, a human orthologue of the mouse tumor suppressor gene, REN^KCTD11^, also antagonizes GLI-mediated transactivation, and its knock-down enhanced HH signaling and cell proliferation in medulloblastoma [42].

**Figure 3:**
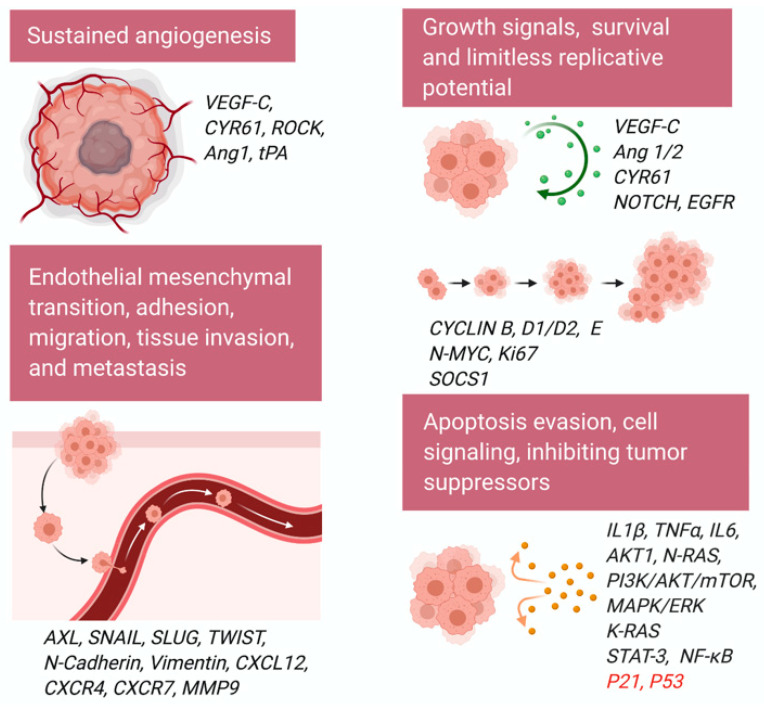
Schematic shows the role of GLI1 and GLI2 in cancer cell proliferation, migration, invasion, cell cycle regulation, angiogenesis, cell signaling, and survival kinases activation. Tumor suppressors downregulated by GLI are shown in red.

GLI transcription factors support tumorigenesis via cell signaling, cell proliferation, angiogenesis, and metastasis [9]. Several studies suggest a crosstalk between RAS-RAF-MEK, PI3K/AKT, and HH/GLI signaling pathways [43,44,45,46]. Not only does endogenous GLI1 activity require intrinsic AKT and MEK signaling function, but AKT1, in combination with N-RAS, enhances nuclear localization and transcription of GLI1 (Figure 3) [43]. Activated K-RAS can also genetically cooperate with activated GLI2 to induce undifferentiated pancreatic tumors [44,46]. A splice variant of p63, ΔNp63α, targets GLI2′s promoter to increase expression and is an important event in osteosarcoma progression during rare breast cancer forms [47]. Mediating this bidirectional transcription response affecting GLI-level transcription determines the HH program’s status and phenotype of cells in relevant tissues. HH pathway genes other than GLI1 include PTCH1, Wnt and TGFβ superfamily proteins, cell cycle proteins (CYCLIN D), and stem-cell marker genes (homeobox protein NANOG and SOX2) [48,49,50]. RAS/RAF/MEK/ERK, PI3K/AKT/mTOR, epidermal growth factor receptor (EGFR), and NOTCH signaling pathways interact at the level of the GLI transcription factors, except for NOTCH, which interferes with the ligand SHH [51,52,53]. 

Constitutive canonical activation of the HH/GLI pathway is a classical representation of aberrant signaling and is typically localized to mutations of *PTCH1* (loss-of-function) and *SMO* (gain-of-function) [54,55]. Inactive HH precursors undergo post-translational modifications to form signal molecules consisting of cholesterol and palmitoyl residues, which enhances ligand activity and diffusion capacity [56]. Non-canonical signaling is often recognized as a deviation from the typical motif of HH signaling, independent of GLI activity, instead of acting through one of the multiple oncogenic pathways such as K-RAS, TGFβ, PI3K-AKT, and PKC-α that target HH genes or are associated with a portion of the HH pathway [54,55,57]. Not only does this provide more prospects for aberrant HH signaling activity, but it also evades existing successful treatments for the canonical pathway such as SMO inhibitor, cyclopamine. In vivo, there may be a combination of canonical and non-canonical HH signaling that is regulated by crosstalk with other intracellular activity. The HH pathway plays an essential role in cell proliferation, differentiation, apoptosis, and migration, and it cross-talks with signaling pathways such as MAPK/ERK, PI3K/AKT/mTOR, EGFR, and NOTCH (Figure 3) [52,58,59,60]. tGLI1 has been reported as a stronger promoter of tumor migration and invasion as compared to GLI1 in glioblastoma and breast cancer [61].

## 5. Involvement of GLI in Cancers

In recent years, the HH signaling pathway has shown significant contributions to tumor initiation, progression to more advanced tumor stages, or low-grade to high-grade tumors [62,63,64,65]. Inappropriate HH signaling plays a role in more than 30% of human cancers [66]. GLI1 overexpression in breast cancer serves as a significant marker of aberrant activation of the SHH pathway driving the formation and progression of breast cancer [67,68,69]. SHH pathway activation promotes mammary epithelial cell mesenchymal transition (EMT) [68,69], and regulates mammary cancer stem cell (CSC) self-renewal, and facilitates angiogenesis [70]. Additionally, inhibiting the GLI1 expression could efficiently mitigate tumor growth and migration and showed its therapeutic potential in breast cancer management [71,72]. Studies reported no significant association between GLI1 expression and histological grade, T stage, clinical stage, and lymph node metastasis in breast cancer. A meta-analysis done in few studies explained GLI1 expression as one of the factors in aggressive biological behavior in breast cancer patients. Further, it elucidated the link between GLI1 expression and prognosis of breast cancer [67,73,74]. GLI1 works downstream of a protein lysine methyltransferase called SET7/9. The knockdown of SET7/9 promotes the proliferation, migration, and invasion of breast cancer cells in vitro and overexpression vice versa [75]. Investigation of the mechanism revealed that overexpression of SET7/9 inhibited GLI1 expression [75], suggesting that GLI1 expression in human breast cancer tissues negatively correlates with SET7/9 expression. Together, these results establish that SET7/9 inhibits oncogenic activities by regulating GLI1 expression in breast cancer [75]. High GLI1 expression in the claudin-low cells and tumors correlates with EMT markers and breast CSCs [76]. GLI1 knockdown in claudin-low cells reduced tumor growth of orthotopic xenografts, and treatment with nuclear factor κB (NF-κB) pathway inhibitor decreases GLI1 expression and protein levels in breast cancer [76].

Inflammatory breast cancer (IBC), a rare (<5%) form of all breast cancers diagnosed in the US, is the most aggressive and lethal form of primary breast cancer targeting young women. IBC is characterized by a higher risk of early recurrence, distant metastases, and spread to the central nervous system than non-inflammatory, locally spread breast cancer [77,78,79]. HH pathway has been studied in the context of pathophysiology in triple-negative breast cancer (TNBC), especially IBC [71]. GLI1 plays a role in proliferation, survival, and migration of IBC cell line SUM149PT, and direct targeting of GLI1 transcription is proposed as a novel and promising strategy for IBC [71]. Mechanisms of crosstalk between HH signaling and TNBC cell survival have potential implications for HH targeting interventions [80]. 

GLI1 was discovered in human glioma [81], and its signaling pathways have been reported in medulloblastoma [82,83,84] and rhabdomyosarcoma [85]. Loss of tumor suppressor SNF5 (SMARCB1) induces aberrant activation of GLI1 in malignant rhabdoid tumors (MRTs) [86]. SMARCB1 is localized to the upstream regions of the transcription start sites of GLI1 and PTCH1 [86]. shRNA mediated knockdown of SMARCB1 upregulated GLI1 and PTCH1 and activated SHH signaling pathway, whereas reexpression of SMARCB1 in MRT cell lines repressed GLI1 expression [86]. 

Pancreatic cancer is one of the most lethal malignancies that require innovative treatments targeting CSCs. Inhibition of HH pathway using GLI inhibitor GANT61 reduced the expression of stem cell marker CD133, and sphere formation of pancreatic cancer cells [87]. The double blockage of HH/GLI and mTOR signaling was also very useful for pancreatic cancer cells [88]. Pancreatic ductal adenocarcinoma (PDAC), the most aggressive human malignancy, thought to be initiated by K-RAS activation, involves transcriptional activation of GLI transcription factors [46]. Ectopic GLI1 activation in the mouse pancreas accelerated K-RAS driven tumor formation, underscoring the importance of GLI transcription factors in pancreatic tumorigenesis [46]. Interestingly, GLI-regulated IκB kinase epsilon (IκBK€) and NF-κB activity were critical for PDAC cell transformation and survival, demonstrating the mechanism of GLI-NF-κB oncogenic activation in pancreatic cancer (Figure 3) [46]. 

Prostate tissues in the embryonic phase require the HH pathway for healthy prostate development that involves ductal morphogenesis through concentrated SHH expression at bud formation sites [35]. SHH null mice exhibit a congenital disorder cyclopia, neural tube malformation, and absence of distal limb structures as developmental defects [89]. Adult tissues require constant SHH activity for epithelial cell turnover, phase regulation, and prostatic growth, branching morphogenesis, and epithelial differentiation [90]. SHH overexpression adds to the metastatic potential with a probable interaction between epithelial and stromal cells and aberrant differentiation of epithelial cells [90]. 

Colorectal cancer is one of the leading causes of cancer death among young adults in the US [91]. Increased levels of GLI1 and GLI2 cooperatively represent a hallmark of unregulated HH activity in colorectal tumor progression. High GLI2 expression stems from an initial genetic mutation acts as a marker for high HH signaling. It predicts poor prognosis and suggests that the potential epigenetic reprogramming is an underlying natural metastatic transition [92]. A novel variation in HH signaling that sustains the colon tumor microenvironment inhibits the repressor form of GLI3 called GLI3R. SUFU, which typically sequesters GLI proteins in the absence of HH ligand binding, can also bind to GSK3β to form the trimolecular complex GLI3/SUFU/GSK3β. This complex activates phosphorylation of GLI3 and subsequent processing/cleavage, which represses the HH pathway [93,94,95,96]. High levels of GSK3β found in colon cancer tissues up-regulate non-canonical HH signaling through maintaining the activator form of GLI3, thus effectively promoting cancer cell survival [93,94,95,96].

HH signaling downstream regulators are over-expressed in both squamous and adenocarcinomatous esophageal cancers [97]. GLI1 binds to the caudal type homeobox 2 (CDX2), responsible for maintaining the intestinal phenotype promoter, activating the site independently of SMO, which supports a non-canonical transition from squamous to columnar epithelium metaplasia in Barrett’s-associated adenocarcinoma cells [98]. A study by Yang et al. found increased HH pathway activation in precancerous esophageal lesions (Barrett’s esophagus and squamous dysplasia), suggesting HH signaling as an early event esophageal cancer development [99]. The same study stated that high levels of SHH, PTCH1, and GLI2 are focally expressed in the epithelium of carcinoma in situ, suggesting potential early screening possibilities for unusual HH signaling activity [99]. Many presentations of gastric cancer are epithelial-derived and manifest in the interstitium or more diffusely as adenocarcinomas. El-Zaatari et al. demonstrated that GLI1 knock-down in mice had reduced expression of a pre-neoplastic phenotype with low IL1β, TNFα, IL6, phosphorylated signal transducer and activator of transcription 3 (STAT3), and proliferative marker Ki67 [100,101]. Studies that introduce GLI2 specific negative regulators in gastric cancer cell lines informed a decrease in cell proliferation, migration, invasion, and an increase in cell apoptosis [102,103].

Previous work in mouse models has shown that GLI2 over-expression in the skin leads to the development of basal cell carcinoma (BCC), with sustained or aberrant upregulation of SHH/GLI cascade for cancer growth [104,105,106]. GLI2 acts as a direct upstream activator of GLI1 via binding to its promoter site and initiates a positive feedback loop in HH signaling.

HH signaling is also involved in ovarian cancer, bladder cancer, endometrial cancer, rhabdomyosarcoma, pancreatic tumorigenesis, non-small-cell lung cancer (NSCLC), melanoma, and hematological malignancies such as acute myeloid leukemia (AML), diffuse large B-cell lymphoma (DLBCL), chronic lymphocytic leukemia (CLL), Hodgkin’s lymphoma (HL), ALK+ anaplastic large cell lymphoma (ALCL), mantle cell lymphoma (MCL), multiple myeloma (MM) and chronic myeloid leukemia (CML) [66,107,108,109,110,111,112,113,114,115]. Dysregulation of SHH, the most potent and widely expressed HH ligand, is characteristic of basal cell nevus syndrome, also known as Gorlin syndrome. [116,117]. Gorlin syndrome is an autosomal dominant neurocutaneous disease mainly triggered by PTCH1 gene mutations [118]. *PTCH1* gene mutations permit SMO transposition and enhance the expression of GLI that drives cell proliferation and tumor growth [118]. SHH dysregulation increases the risk of developing BCC, medulloblastoma, and meningioma. Activating or loss of function mutations in the HH pathway genes SMO and PTCH1 occur in human gastric tumors [119], sporadic BCC [120], medulloblastoma, nevoid basal cell carcinoma syndrome (NBCCS), and colorectal cancer [121]. 

## 6. GLI and Hallmarks of Cancer

### 6.1. Angiogenesis

Initial stages in preparing the tumor microenvironment include a breakdown of the vascular membrane and extracellular matrix (ECM), proliferation, and migration of endothelial cells (Figure 3). High expression of a common factor initiating angiogenesis, vascular endothelial growth factor-C (VEGF-C), is often correlated to increased vascularity in GLIoma formation [122]. There is a potential link between elevated HH signaling and increased transcription of VEGF-C (Figure 3). Another factor, cysteine-rich angiogenic inducer 61 called CYR61, is an ECM associated signaling molecule that promotes the adhesion of endothelial cells through interaction via integrin. CYR61 contains pro-angiogenic characteristics at the embryonic and wound healing properties at adult levels [123,124]. SHH-expressing breast cancer cells exhibit unregulated levels of CYR61 at the transcript level. A GLI1 binding site exists upstream of the CYR61 transcription start site, and mutation or deletion of this GLI1 binding site results in diminished activity of the CYR61 promoter in the presence of GLI1 [125]. SHH influences angiogenesis in endothelial cells directly through the Rho/RhoA and Rho kinase (ROCK) signaling pathway [126]. SHH induces the expression of matrix metalloproteinase 9 (MMP9) and osteopontin (OPN) [126]. MMP9 is involved in the degradation of the ECM [126]. OPN, also known as bone sialoprotein I (BSP1), is a highly chemotactic glycoprotein involved in bone remodeling, bone mineralization, immune cell activation, and anti-apoptosis in various cancers and viral infections [126]. Genetic ablation of the tissue plasminogen activator (tPA) in mouse brain endothelial cells (MBECs) impaired tube formation and downregulated VEGF and angiopoietin 1 (Ang1). Addition to rh SHH to tPA^−/−^, MBECs partially restored the tube formation and upregulated Ang1, but not VEGF, although rh SHH increased VEGF and Ang1 expression on wild-type MBECs. Complete restoration of tube formation in tPA^−/−^MBECs was observed when both exogenous SHH and tPA were added, demonstrating the role of SHH-induced in vitro cerebral angiogenesis during the brain repair after stroke [127].

### 6.2. Epithelial to Mesenchymal Transition (EMT)

Downstream effects of GLI family activation such as EMT transform adult tissues to a mesenchymal-like state, losing polarity and adhesive properties while gaining migratory characteristics that encourage the invasive nature of cancer cells (Figure 3) [128]. This includes stem-cell features such as non-adherent growth, changes in the expression of cell-surface glycoproteins, and surface marker expression of stem cells [129]. The net structural transition of epithelial cells is from an attachment to the underlying ECM to migration into the matrix [130,131]. HH signaling induces the trans-differentiation of epithelial cells by decreasing E-Cadherin expression levels, increasing β-catenin and vimentin expression, tissue invasion, migration, and colony formation; it also transcribes necessary EMT regulators such as SNAIL, SLUG, and TWIST via GLI1 in adult human placentas [132]. In mouse models of gastric adenocarcinoma, EMT seems to be a particular phenotype of activated GLI2 [133]. These metastatic cells undergo rapid proliferation and differentiation typical of tumor heterogeneity and become locally invasive through basement tissues and manifest as tumor islands in the gastric mucosa—features only present with activated GLI2. Normal E-cadherin levels maintain cell attachment and layered phenotype of the villous cytotrophoblast. In contrast, EMT-induced reduction of E-cadherin and redistribution at cell junctional regions promotes loosened cell-to-cell connections and apicobasal polarity [132].

Further evidence in human melanoma cells suggests that GLI2 is directly responsible for turning off the E-cadherin gene (*CDH1*) expression during EMT in addition to enhancing the transcription of other EMT activators [134]. While this mesoderm expression process is an essential precursor to the differentiation of multiple tissue types and generation of organs in embryogenesis, it also enables a microenvironment of asymmetrical cell division leading to macroscopic metastases in adult tissues. Various target molecules regulate the proliferation pathway of HH signaling (Figure 3). Ectopic expression of Rab23 acts as a negative regulator of HH signaling at the level of GLI1 and GLI2 mRNA expression [135,136]. Interestingly, Rab23 upregulates the repressor form of GLI3, which endogenously inhibits the HH signal cascade [137]. EMT cells increase breast cancer metastasis via paracrine GLI activation in neighboring tumor cells and triggering HH/GLI signaling cascade [138]. GLI3 repressor (GLI3R) inhibits HH signaling, and GLI3R is essential for response to SMO antagonist glasdegib in AML [139]. GLI3 is silenced in most AML patients [139]. GLI3R represses AML growth by downregulating AKT expression [140]. GLI3R plays an essential role in SMO-independent HH signaling in AML and suggests that GLI3R could serve as a potential biomarker for patient selection in SMO antagonist clinical trials. GLI3 inactivation results in additional digit formation in vertebrates [140]. GLI3 works as a negative modulator of the proliferative expansion of digit progenitors by restricting the G1 to S cell-cycle transition by regulating CDK6 and constrains S phase entry of digit progenitors [140].

### 6.3. Cell Cycle

Aberrant HH signaling triggers a series of vasculogenic and angiogenic processes that endorse tumorigenesis and tumor growth in adult tissues. Several proposed that feedback loops regulate HH pathway activity (Figure 3). IFN-γ/STAT1 signaling has tumor suppressor function and is inactive in at least one-third of all melanoma and lung adenocarcinoma cell lines in mice [141]. SOCS1, an IFN-γ/STAT1 inhibitor, is activated by HH signaling pathway itself to create a negative feedback loop and a downstream target of GLI1 and GLI2, which upregulates its transcriptional activity and subsequently relieve the IFN-γ/STAT1 form obstructing tumor growth [142]. Common cell cycle genes turning on via the HH pathway include *CYCLIN D* and *E*, which are necessary to induce G1-to-S transition in the cell cycle, and *CYCLIN B*, which activates mitosis promoting factors (Figure 3). HH signaling opposes normal stimuli for epithelial cell cycle arrest such as *P21* and inhibits the *P53* tumor suppressor gene [143,144]. 

### 6.4. Migration/Adhesion/Invasion/Metastasis

Mechanisms of HH induced metastasis are understood at a broad level, but specific phases remain under deliberation. Upregulation of G-protein coupled receptors (GPCRs), chemokines CXCR4 and CXCR7 enhance the directional migration of breast cancer cells in lung metastasis by way of CXCL12 [145], which is a highly-secreted signaling protein of metastatic organs [146]. HH pathway upregulation of GLI1 enhances the CXCL12 induced migration of cancer cells [147]. Administering CXCR4 specific inhibitors or knockdown treatments suppress cancer cell migration patterns in breast and pancreatic cancer in mouse models and in vitro [145]. Part of the migration effectiveness depends on the EMT-programmed loss of cell–cell adhesion that leads to motility. GLI1 activates EMT by inducing SNAIL expression. SNAIL causes fibroblastic conversion, malignant transformation, and loss of a cell–cell adhesion molecule called E-cadherin [148]. Once the adhesive properties of the membrane are compromised, metastatic cells invade the matrix. The EMT program upregulates the expression of essential invasion factor AXL, which is required to maintain SNAIL, SLUG, and TWIST expression in pancreatic adenocarcinoma cells [149]. Interestingly, these same factors potently induce AXL expression, contributing to a positive feedback loop that continues the proliferation of malignant mesenchymal tumor cells [149]. GLI2 knockdown studies supporting its role in migration and invasion in osteosarcoma, prostate cancer, and hepatocarcinoma cell lines underscores the metastatic potential of GLI [150,151].

GLI2 expression directly enhances tumorigenesis in a model of myofibroblastic cells representing reactive stromal prostate cancer cells [152]. There is still some controversy regarding the exact model of hormone signaling in prostate tissues: paracrine versus autocrine. Paracrine signaling from the epithelium supports stromal differentiation during prostate development and sustains the stroma in the adult prostate [153,154]. In xenograft tumors, SHH is localized to the prostatic epithelium, while GLI1 mRNA is localized to the stromal compartment suggesting paracrine HH signaling [155]. Other studies support the idea of a shift to autocrine SHH signaling during pathogenesis and progression of prostate carcinoma [156]. There may be some interplay between these two forms of hormone activity in vitro, at least during the introduction of tissue metastasis, if not during the tumor progression.

## 7. HH Signaling Pathways during Infections and Viral Malignancies

Besides controlling processes involved in embryogenesis, there are broader implications of HH signaling. Recent studies revealed that aberrant activation of HH signaling leads to pathological consequences [157]. Viruses enter the host by interacting via their surface proteins, hijacking host replication machinery, targeting several signaling pathways concurrently, and overturning host immune mechanisms and evolutionary benefit from the host cell machinery. The virus makes copies of itself and spreads those copies to new hosts. Many viruses have evolved to stimulate host HH signaling to control their life cycle and pathogenesis [157,158]. There is considerable speculation of pathogens to use HH signaling to regulate their life cycle.

**Figure 4:**
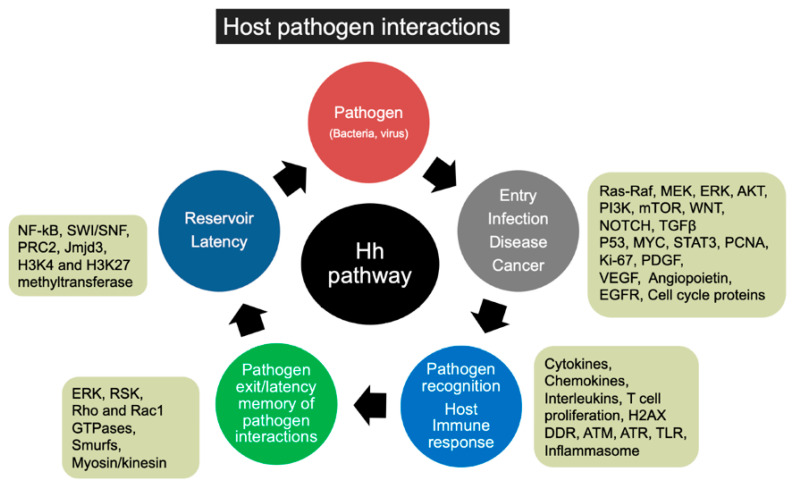
Schematic shows various stages and consequences of host-pathogen interactions. It also shows multiple signaling cascades, and transcription factors are demonstrated to be regulated by the HH pathway. Signaling molecules and transcription factors regulated by HH signaling could contribute to pathogen (virus or bacteria) entry, infection, pathogenesis (angiogenesis, fibrosis, inflammation), and activation of host foreign recognition network, host immune response, pathogen exit/elimination, and pathogen latency in the host.

Here, we discuss influenza-A, hepatitis B virus (HBV), hepatitis C virus (HCV), human immunodeficiency virus (HIV), human papillomavirus (HPV), Merkel cell polyomavirus (MCPyV), human T-cell leukemia virus, type 1 (HTLV-1), Epstein–Barr virus (EBV) and Kaposi’s sarcoma herpesvirus (KSHV) infection to highlight the plausible involvement of HH pathway players in the viral life cycle. We discuss the possible role of HH signaling in viral latency, virus reservoir maintenance, entry, replication, pathogenesis, infected cell proliferation, infection progression, pathogen recognition in the host, and the immune response of the host, exit of the pathogen and memory of the host immune mediators (Figure 4). 

HIV [159,160], HCV [161,162], HBV [163], EBV, and KSHV [164,165,166] utilize VEGF to maintain the vascularity of the tumors and infected cell proliferation. HH signaling plays an indispensable role in vascular development in mouse embryos via VEGF and NOTCH signaling [167]. SHH regulates the angiogenic VEGF and angiopoietin (Ang 1 and Ang 2) in astrocytes by activating the nuclear receptor subfamily 2 group F member 2 (NR2F2) transcription factor [168] (Figure 3). HIV [169], HCV [170], HBV [171,172], EBV and KSHV [173,174,175,176] infection promotes angiopoietin expression. Maturation of VEGF-induced new vessels in the cornea involves a platelet derived growth factor (PDGF)-SHH axis that mediates PDGF-BB–mediated smooth muscle cell (SMC) migration by inducing ERK1/2 and PI3Kγ activation [177]. Elegant studies described that KSHV utilizes host PDGF receptor-α (PDGFRα) to drive its tumorigenesis and is prominently active in murine and human AIDS-associated-Kaposi’s sarcoma (AIDS-KS) development [178]. Exclusively blocking PDGFRα signaling could impede murine KS tumor formation [178]. There is a strong correlation between the HH pathway and hypoxia and hypoxia-inducible factor 1 (HIF1). Hypoxia induces upregulation and secretion of SHH in the human pulmonary arterial smooth muscle cells (HPASMCs) [179]. SHH expression depends on HIF1 in the HPASMCs, and hypoxia stimulates GLI1 nuclear translocation [179]. Overall, hypoxia induces HPASMC proliferation, and the HH signaling pathway regulates apoptosis in the HPASMCs subjected to hypoxia [179]. HIV [180], EBV [181], and KSHV [182,183,184,185,186], HCV [187], and HBV [182] utilize the host HIF1 factor for redox signaling during their life cycle.

HH signaling is a potential target for manipulating viruses, as this pathway plays a fundamental role in cell survival and proliferation [188,189,190]. The HH target gene PTCH1 is one of the critical host genes involved in influenza infection and favors viral dissemination [191,192]. Nonstructural protein 1 (NS1), one of the multifunctional proteins, limits the lung’s injury to preserve the viral habitat in the host. HH signaling plays a significant role in branching morphogenesis during pulmonary development [193]. Immunohistochemical analyses have indicated increased TGFβ1 expression at the epithelial site of fibrosis, such as cryptogenic fibrosing alveolitis and bronchiectasis. SHH antagonizes TGFβ1 by inducing epithelial repair [194]. Patients with scarring in distal lung secondary to bronchiectasis show prominent perinuclear staining for SHH, thus relating the SHH staining to the level of injury [195].

Moreover, the HH receptor, PTCH1, is expressed in infiltrating and circulating lymphocytes (CD4 and CD8) at both protein and mRNA levels. It is evident that immune cells are equipped to respond to the HH ligand secreted from the inflamed area [196]. HH signaling thus plays an essential role in repairing damaged lung tissue by remodeling the epithelium directly or in concurrence with activated immune cells and regulates the communication of immune cells. Therefore, controlling the HH response may help the host to avoid the harmful consequences of viral infection. A similar study on larval *Drosophila melanogaster* has shown that the effect of the NS1 on the HH pathway is conserved between the species. A point mutation (A122V) was identified in *Drosophila,* which could reduce the HH-dependent activity of the NS1 in flies and transfected cells [191]. However, the same mutation, when incorporated into a mouse-adapted influenza-A virus, intensified the expression of some HH targets in the mouse lung and significantly accelerated lethality. No mutation at position 122 of NS1 has been recognized in any influenza strains, and thus NS1 protects the host. This muting of HH signaling may be utilized to diminish the harmful effects that could be caused by HH signaling to ensure optimal viral maturation before dissemination. 

HIV [197], HCV [198], and HBV [199], EBV [200], and KSHV [201,202,203] are well-known to program their genes selectively to modulate the DNA damage response (DDR). KSHV manipulates DDR either via activation of ataxia telangiectasia mutated (ATM) pathway or by phosphorylating factors associated with the DDR, such as tumor suppressor protein P53 [201,202,203]. Interestingly, canonical HH signaling or ectopic expression of GLI1 causes genomic instability and cancer predisposition by faulting the S-phase checkpoint, DNA repair mechanisms, and inhibiting DNA double-strand breaks (DSBs)-mediated DDR [204]. ATM and Ataxia telangiectasia and RAD3 related (ATR) protein serine-threonine kinases involved in cell-cycle checkpoint signaling are regulated by HH signaling [205]. GLI inhibitor GANT61 induces DNA damage. GANT61 treatment increases the nuclear foci of γH2AX and activates ATM and Chk2 in human colon carcinoma cells [206].

HH signaling also contributes to the epigenetic regulations. HH target genes are poised and are marked by an active (H3K4me3) and a repressive (H3K27me3) mark maintained by the H3K27 methyltransferase polycomb repressive complex 2 (PRC2) [207]. SHH induction recruits the Jumonji domain-containing protein D3 (Jmjd3), a histone H3K27 demethylase, which dislodges PRC2, removes H3K27me3, and enlists the Set1/MLL H3K4 methyltransferase complex to initiate gene expression [207]. DNA viruses exploit epigenetic regulation in maintaining viral episomes through the generation of chromatin, controlling viral gene transcription and replication or evading of the host innate immune response [208]. Excitingly, HIV [209], HCV [210], HBV [211,212], EBV [213,214] and KSHV [215,216] employ host methyltransferases, histone modification enzymes, histone acetylases, deacetylases, and demethylases, etc., for their chromatin remodeling, reactivation from latency, and viral life cycle propagation. 

HBV and HCV are associated with liver cirrhosis and hepatocellular carcinoma (HCC) worldwide [217]. Pereira et al. showed increased hepatocyte production of HH ligands in patients with chronic HBV and HCV infection [218]. HH pathway activation often occurs during fibrogenic repair of liver damage due to chronic viral hepatitis [218]. HH-responsive cells facilitate hepatocarcinogenesis and disease advancement in chronic viral hepatitis [218]. Another study further confirmed the increased expression of HH targets in a GLI-dependent manner when liver cells were treated in vitro with the whole HBV replicon or with serum from HCV-infected patients [219]. This caused pro-fibrotic effects [219]. Hepatitis B virus x (HBx), one of the HBV viral proteins, increases GLI1 protein nuclear accumulation [220]. HBx protein stimulates the HH-GLI activation through protein stabilization and nuclear localization of GLI1 in liver cancer cells while the exact role of GLI1 protein translocation for these viral activities remains to be discovered [220]. Blocking HH signaling delayed hepatocarcinogenesis induced by HBx protein (Table 1) [221]. HH signaling in liver cells is associated with increased permissiveness for HCV replication and viral production [222]. 

The HH pathway maintains and controls stem cells and policies of hematopoiesis and lymphopoiesis to maintain immune cells [223,224,225,226]. Increased GLI1 expression in EBV infection decreases the level of human leukocyte antigen (HLA) and helps the virus to escape cytotoxic T cell recognition [227]. The HH pathway is activated in EBV derived nasopharyngeal carcinoma (NPC) tissue, NPC-derived cell lines, and in EBV infected epithelial cells [228]. HIV-related nephropathy presented increased expression of GLI and associated proteins of the HH pathway [217]. EBV latent membrane protein 2A (LMP2A) utilizes Gli1 to downregulate HLA in gastric cancer cells [229]. The same was observed in a human podocyte cell line infected with HIV [217]. These aberrant pathway activities decrease host defense by increasing proliferation and migration markers, loss of kidney filtration barrier function, and increased permeability. All these changes observed could seemingly boost viral dissemination and increase host infectivity [217]. HIV infection is linked to the elevated level of immunoregulatory cytokine TGFβ1 that leads to the suppression of host protective immune responses [230]. GLI2 regulates TGFβ1 at the transcriptional level in human CD4+ T cells during HIV infection [230]. Human SMO inhibits HIV-1 replication and disease [231]. The role of the HH pathway was tested in HIV-induced EMT, which is critical for the progression of kidney injury [217]. The blockade of the HH pathway with GANT 58 (GLI antagonist, a specific blocker for GLI1-induced transcription) treatment could dramatically decrease HIV-induced podocyte EMT, permeability, and fibrosis of the kidney (Table 1) [217].

HH regulates the expression of OPN in nonalcoholic steatohepatitis-related liver fibrosis as GLI directly interacts with the GLI-binding sites in the OPN promoter [232]. A decrease in either HH signaling or OPN decreases fibrosis [232] as OPN is a direct transcriptional target of the HH pathway [232]. Treatment with GLI1 inhibitor or cyclopamine plays a protective role in a mouse model of renal fibrosis following injury and expressing the activated HH signaling pathway [232]. Some viruses may promote HH signaling to induce fibrotic damage to ensure viral spread [157,217,233]. Other pathogens, such as the influenza virus, limit fibrotic tissue formation, thereby allowing more time for replication, maturation, and dissemination of infection [157,191]. However, to maintain progeny, these viruses make sure to support viable hosts available for reinfection. 

Merkel cell polyomavirus (MCPyV) is detected in approximately 80% of Merkel cell carcinoma (MCC), an aggressive neuroendocrine skin cancer mostly occurring in the elderly. Reactivation of HH signaling later in life can cause tumors. 29 MCPyV-positive and 21 MCPyV-negative MCCs were stained for SHH, IHH, PTCH1, SMO, GLI1, GLI2, and GLI3 and expression of the HH signal pathway players associated with MCPyV infection and prognosis of MCC [234]. 

HPV oncogenes (E6/E7) and estradiol, major etiologic factors associated with cervical cancer, could induce GLI activity in the cervix and the skin in mice [235]. Treatment with a putative novel HH inhibitor itraconazole, could not diminish HH signaling, but it reduced growth at an early stage of cervical carcinogenesis (Table 1) [235]. While these studies suggested the possible involvement of HH signaling in cervical carcinogenesis, the mechanism is not known [235]. GLI1 and GLI2 overexpression serve as a prognostic factor for overall and disease-free survival in patients with locally advanced HPV negative head and neck cancer undergoing surgery and postoperative radiotherapy [236]. The human poliovirus receptor CD155 gene acts as a transcriptional target of SHH and is activated by SHH in neuroectodermal tumors [237].

Tumor cells of Hodgkin lymphoma (HL) are derived from mature B cells. The lineage infidelity of Hodgkin/Reed-Sternberg cells (HRSs) often causes diagnostic problems as HRS markers are also favorable for follicular dendritic cells (FDCs). Investigation of the expression of FDC markers in HL and anaplastic large cell lymphoma (ALCL) revealed GLI3, fascin (actin-bundling protein found in membrane ruffles), and TUBB3 (a member of the beta-tubulin protein family) as the most sensitive markers, which were diffusely positive in HL [238]. A recent study from our lab reported the increased expression of GLI1 in KSHV infected primary effusion lymphoma (PEL) cells [239].

Anti-inflammatory lipoxin A4 treatment in PEL cells downregulated GLI1 expression [239]. The decrease in GLI1 and PTCH1 expression was not dependent on SHH ligand activity, as we did not observe any significant change in SHH expression in the solvent or lipoxin A4 treated PEL cells. Interestingly, we found increased phosphorylation of GLI1 at Thr 1074 and decreased phosphorylation of AKT/mTOR proteins in lipoxin A4 treated PEL cells. GLI1 phosphorylation at Thr 1074 led to the degradation of GLI1 through 5′ adenosine monophosphate-activated protein kinase (AMPK) activation (through the phosphorylation of AMPK at Thr 172) and reduced the oncogenic potency of GLI1 by preventing the transcription of target genes GLI1 and PTCH1. We are testing the therapeutic potential of GLI inhibitors in KSHV related cancers, including KS and PEL.

HTLV1 expression is activated by the interaction of a viral transactivator protein, TAX, and cellular transcription factor, CREB (cyclic AMP response element-binding protein), binds to the long terminal repeat (LTR). The human homolog of a member of the GLI oncogene family, GLI2 (also termed hGLI2), helps HTLV1 infection progression as the simultaneous binding of hGLI2 and CREB seems critical for TAX protein to activate transcription [240]. HIV [241], HCV [242], HBV [243,244], EBV [245], and KSHV [246] utilize MYC and cellular STAT3 for infected cell proliferation, the persistence of herpesviruses latency, and inhibition of viral reactivation. HIV [247], HCV [248], HBV [249], EBV and KSHV [250,251] infected cells secrete cytokines and chemokines to regulate viral pathogenesis, evade host immune response (Toll-like receptors; TLRs and inflammasome), angiogenesis, and selectively chemoattracts T cells, activation and migration of immune cells. HIV1 envelope protein R5 gp120 exposure to immature monocyte-derived DCs (MDDCs) resulted in the CCR5-dependent production of interleukin-6 (IL6) cytokine via mitogen-activated protein kinase (MAPK)/NF-κB pathways [252]. IL6 could activate STAT3 by an autocrine loop, further contributing to IL6 secretion [252]. HH pathway has been demonstrated to act synergistically with interleukin-6 to drive the growth of basal cell carcinoma via STAT3 activation [253]. Mechanistically, IL6 and HH/GLI signaling integration occur at the level of cis-regulatory sequences by co-binding of GLI and STAT3 to common HH-IL6 target gene promoters and HH-IL6 pathway combinatorial blockade could efficiently arrest cancer growth in BCC patients [253]. These transcription factors, signaling pathways mediating IL6 induction have not been tested in the context of viral infections, which depend significantly on the IL6 for survival and progression of infection such as KSHV [254], H1N1 influenza A infection [255], Pneumovirus infection; closely related to a respiratory syncytial virus [256], Hepatitis B Virus [257], and EBV [258].

HH signaling is also regulated by MAPK and NF-κB cascade and HH/GLI1, MAPK (KRAS-MEK-ERK) cascade and NF-κB cooperate to regulate growth and cell proliferation [259], and apoptosis resistance in many tumors [260]. These studies provide a potential link that could be playing an important role in the lifecycle of many viruses, which activate MAPKs and NF-κB in host cells upon binding, entry, or during the stage of viral gene expression. Similar to MAPK, ERK, PI3K, and NF-κB, HH/GLI pathway plays an important role in the induction and sustenance of Rho-GTPases and stimulates cell migration [261,262]. HIV [263], HCV [264], HBV [265], EBV [266] and KSHV [267,268,269]. These viruses utilize signaling pathways such as ERK, RSK, PI3K, Rho, and Rac1 GTPases for cell cycle progression, viral entry, cellular transformation, expression of viral genes, and the establishment of infection. HIV [270], HCV [271,272], HBV [273,274,275], EBV, and KSHV [276,277,278,279] exploit host AKT, PI3K, mTOR signaling pathways for the evasion of apoptosis, infected cell survival, and proliferation, viral replication, production, vesicle formation, intracellular motility and activation of transcription factors. HIV [280], HCV [281], HBV [282], EBV [283], and KSHV [269] utilize myosin/kinesin for viral transmission, virion transcytosis, virus entry, intracellular viral transport, and formation of highly metastatic and invasive tumors with the leading edge. 

Not only viruses, but bacterial infections such as *Salmonella enteritidis* ST183 [284] *Escherichia coli* [285], and *Helicobacter pylori* [286] are also inclined to use the HH signaling pathway to control the infection progression and the infected cell microenvironment [284,285,287,288] (Figure 4). *Helicobacter pylori* infection mediated inflammation and repair process involving macrophage recruitment activates transcription factor NF-κB and also upregulates HH proteins [286].

## 8. GLI Inhibition and Implications as Anticancer Therapeutics

Currently, small molecule modulators such as SMO and GLI1 inhibitors of HH signaling have been used in basic research to detect links between signaling and specific phenotypes of interest [289]. Few inhibitors are now in use to treat certain malignancies associated with viral infections (Table 1), BCC and certain leukemia, whereas many others are still in clinical trials [290]. Till now, vaccines and antivirals, which are specific to rapidly mutating viral proteins, have been used, but now with the use of HH inhibitors, a broad spectrum of strains can be targeted. Since the HH signaling controls many critical cellular processes, simultaneously, there is a need to target a specific pathway component selectively. This process may require combinatorial drug usage targeting different HH-dependent processes, which may further regulate signaling in the infected cells. An in-depth understanding of the precise mechanism by which viral factors would interact with the HH pathway players would prove beneficial for targeted therapies.

Aberrant HH signaling has been found responsible for chemo-resistance in aggressive cancers [291,292]. Much work has already shown the worth of the GLI family as emerging targets for cancer therapy [293]. GLI1 inhibitors have demonstrated the broadest therapeutic potential so far as a target in advanced and metastatic tumors [293]. GLI1 expression has been used as a potential prognostic factor for survival in bladder and colon cancer [294]. Inhibition of upstream MEK1/2-ERK1/2 activity with U0126 inhibitor in human HT29 colon cancer cells suppress GLI transcriptional activity and subsequent protein expression [96]. GLI1 also supports a correlation between low expression and more prolonged survival in patients with oral squamous cell carcinoma [295]. Breast tumor cells upregulate GLI2 expression during bone metastasis, stimulating bone resorption, activating TGFβ, and subsequent tumor proliferation [296]. Aberrant HH signaling activation promotes the growth of BCC, medulloblastoma, colorectal cancer, and small lung cell cancer [289]. This has led to a large repertoire of small molecule inhibitors developed for the treatment of cancers dependent on the HH pathway. 

Among SMO inhibitors, GDC-0449 and LDE-225 are in the clinic for the treatment of advanced BCC with aberrant HH activity due to loss of the functional allele of PTCH [297,298] The steroidal alkaloid, cyclopamine, has progressively shown therapeutic potential as an inhibitor for HH signaling. The mechanism of cyclopamine action suggests an interaction with the SMO heptahelical bundle, promoting a protein conformation through small endogenous molecules rather than direct protein-protein interaction [292,299]. In GLIoma-derived neurospheres, cyclopamine blocked inhibited overall growth rate by 30–70% [291,300]. Vismodegib (trade name Erivedge) is another SMO inhibitor that effectively terminates HH signaling. Vismodegib is also an FDA approved treatment for basal cell carcinoma in adult patients [301]. There is less support for cyclopamine and its analogs as therapies in cancers with bone metastases such as IBC, wherein GLI2 needs to be inhibited further downstream from the HH receptors [302]. LDE-225 (Erismodegib/Sonidegib/Odomzo), HH pathway inhibitor, received FDA approval to treat cancer patients [303,304]. Many phase I and phase II trials for Erismodegib as a monotherapy and in combination are underway, treating malignancies including advanced gastroesophageal adenocarcinoma, small cell lung cancer, myelofibrosis, advanced/metastatic HCC, and relapsed medulloblastoma [305,306].

IPI-926 (Saridegib), CUR61414 binds to SMO and prevents its activation, BMS-833923/XL139 (binds SMO), PF-04449913; Glasdegib (SMO inhibitor), PF-5274857 (SMO antagonist), TAK-441 (SMO inhibitor), LY2940680; Taladegib (SMO antagonist), MRT-92 (anti-SMO activity by blocking several overlapping sites of the SMO transmembrane domain), Jervine (binds to SMO and preventing its conversion to an active state), RU-SKI 43 (SHH Inhibitor), and SHH Monoclonal Antibody 5E1 (SHH Inhibitor) is SMO or SHH Inhibitor in clinical trials [305,306].

A well-known small molecule inhibitor, GANT61, reduces GLI1, GLI2, and PTCH1 mRNA expression in human colon carcinoma cell lines [96]. GANT61 appears to be a more potent treatment in colon carcinoma cell lines than its upstream-acting counterpart, cyclopamine. Another study found that cells treated with GANT61 accumulated at G1/S followed by early S and finally underwent cell death at 48h, and cyclopamine treated cells demonstrated minimal effects on cell cycle distribution or cell death [96]. Arsenic Trioxide (ATO) is an FDA approved inhibitor of GLI1 and GLI2 transcription factors for acute promyelocytic leukemia treatment [303]. In esophageal adenocarcinoma (EAC), elimination of S6K1 activation by mTOR pathway inhibitor enhances the killing effects of the HH pathway inhibitor [52]. In EAC, the activated mTOR/S6K1 pathway promoted GLI1 transcriptional activity and oncogenic function through S6K1-mediated GLI1 phosphorylation at Ser84, which released GLI1 from its endogenous inhibitor, SUFU. 

SHH pathway is highly activated in pancreatic CSCs and plays an essential role in cancer initiation, progression, and metastasis [88]. Sanguinarine, an anti-inflammatory and antioxidant compound isolated from the roots of Macleaya cordata and M. microcarpa, could inhibit cell proliferation, colony formation, self-renewal capacity, and induced apoptosis in human pancreatic CSCs through oxidative damage. Sanguinarine inhibited the SHH-GLI pathway, leading to modulation of GLI target genes in pancreatic CSCs. Sanguinarine inhibited the binding of NANOG with promoters of CDK2, CDK6, FGF4, c-MYC, and OCT4, suggesting the direct involvement of NANOG in the cell cycle, pluripotency, and self-renewal of pancreatic CSCs via SHH-GLI-NANOG pathway [304]. Aspirin (acetylsalicylic acid, ASA), the most widely used drug for its analgesic, antipyretic, and anti-inflammatory properties, has a broad-spectrum antitumor effect in pancreatic cancer, colorectal adenomas, hepatocellular carcinoma, etc. [307]. Recently, aspirin was reported to exert its antineoplastic property in glioma by abrogating the tumorigenic effect of the SHH/GLI1 signaling pathway, especially sensitizing the malignant glioma cells resistant to temozolomide (TMZ) therapy [307].

## 9. Perspectives

GLI transcription factors interact with multiple signaling pathways. Therefore, many combination treatment modalities are under clinical trials, including PI3K inhibitor (BKM120 or BEZ235) and SMO inhibitor (LDE225), an mTOR inhibitor and GANT61, simultaneous targeting of GLI1 and HIF2α. LDE225/Sonidegib and GDC-0449/Vismodegib SMO inhibitors are FDA approved for treating basal cell carcinoma. Clinical trials are ongoing to evaluate the efficacy of this novel class of targeted therapy in various malignancies. The paracrine mechanism plays an essential role in upregulating the HH signaling in a tumor animal model, suggesting the need to consider the stroma-tumor interactions in HH/GLI signaling in the experimental models [105]. HH signaling and GLI transcription factors gain attention and interest in host-pathogen communications and are emerging as a pathogenic target that calls for further investigation. Recent studies highlight the role of HH/GLI signaling in the modulation of the anti-tumoral immune response. These studies have uncovered the functions of HH/GLI in immunosuppression and the production of immunosuppressive cytokines. This exciting development and novel research findings warrant future evaluation of the combination of cancer immunotherapy with HH/GLI inhibitors.

## Figures and Tables

**Table 1:** Therapeutic strategies against viral diseases.

Hh Inhibitors	Disease	Mechanism of Action
Vismodegib *(GDC-0449)*	HBV and HCV	Decreases liver fibrosis in human Decreases tumor formation in a mouse model of fibrosis-associated HCC
		Reduces the growth of HBV X-expressing tumor xenografts in nude mice and HCC formation in transgenic mice expressing the HBV X protein
GLI inhibitors: GANT-61	EBV and EBV linked Nasopharyngeal cancer	Reduces the pro-fibrotic effects Inhibits autophagy in HCV-exposed fibroblasts
		Reduces tumor-sphere formation in several EBV-infected cell lines
	HPV	Decreases the proliferation of Human Papilloma Virus-derived cervical cancer cells
GLI agonist	HIV	Targeted against local uninfected environment
Smoothened agonist		Targeted against HIV infected Cells. Limits viral niche.

## Abbreviations

ALCL	Anaplastic large cell lymphoma
ATO	Arsenic Trioxide
CDH1	E-cadherin gene
CSCs	Cancer stem cells
CREB	cyclic AMP response element-binding protein
CYR61	Cysteine-rich angiogenic inducer 61
DHH	Desert HH
EBV	Epstein–Barr virus
ECM	Extracellular matrix
EMT	Epithelial to Mesenchymal Transition
FDCs	Follicular dendritic cells
GLI	Glioblastoma-associated-protein
GSK3β	Glycogen Synthase Kinase 3 Beta
HH	Hedgehog
HTLV-1	Human T-cell leukemia virus type 1
HBV	Hepatitis B
HCV	Hepatitis C
IBC	Inflammatory Breast Cancer
IHH	Indian HH
IL6.	Interleukin-6
KSHV	Kaposi’s sarcoma herpesvirus
LTR	Long terminal repeats
MAPK	Mitogen-activated protein kinase
MCPyV	Merkel cell polyomavirus
NSCLC	Non-small-cell lung cancer
NLS	Nuclear localization signal
NES	Nuclear export signal
PTCH1	Patched1
SMO	Smoothened
SHH	Sonic HH
SUFU	Suppressor of fused
VEGF-C	Vascular endothelial growth factor-C

## References

[1] Kobayashi T., Yasuda K., Araki M. (2010). Coordinated regulation of dorsal bone morphogenetic protein 4 and ventral Sonic hedgehog signaling specifies the dorso-ventral polarity in the optic vesicle and governs ocular morphogenesis through fibroblast growth factor 8 upregulation. Dev. Growth Differ..

[2] Baron M. (2001). Induction of embryonic hematopoietic and endothelial stem/progenitor cells by hedgehog-mediated signals. Differentiation.

[3] Ahn S., Joyner A.L. (2005). In vivo analysis of quiescent adult neural stem cells responding to Sonic hedgehog. Nature.

[4] Awasthi A., Woolley A.G., LeComte F.J., Hung N.A., Baguley B.C., Wilbanks S.M., Jeffs A.R., Tyndall J.D.A. (2013). Variable expression of GLIPR1 correlates with invasive potential in melanoma cells. Front. Oncol..

[5] Wang K., Pan L., Che X., Cui D., Li C. (2010). Sonic Hedgehog/GLI1 signaling pathway inhibition restricts cell migration and invasion in human gliomas. Neurol. Res..

[6] Lo H.W., Zhu H., Cao X., Aldrich A., Ali-Osman F. (2009). A novel splice variant of GLI1 that promotes glioblastoma cell migration and invasion. Cancer Res..

[7] Gailani M.R., Bale S.J., Leffell D.J., DiGiovanna J.J., Peck G.L., Poliak S., Drum M.A., Pastakia B., McBride O.W., Kase R. (1992). Developmental defects in Gorlin syndrome related to a putative tumor suppressor gene on chromosome 9. Cell.

[8] Farndon P., Del Mastro R., Kilpatrick M., Evans D. (1992). Location of gene for Gorlin syndrome. Lancet.

[9] Kasper M., Regl G., Frischauf A.-M., Aberger F. (2006). GLI transcription factors: Mediators of oncogenic Hedgehog signalling. Eur. J. Cancer.

[10] Epstein E.H. (2008). Basal cell carcinomas: Attack of the hedgehog. Nat. Rev. Cancer.

[11] McMahon A.P. (2000). More surprises in the Hedgehog signaling pathway. Cell.

[12] Varjosalo M., Taipale J. (2006). Hedgehog signaling. J. Cell Sci..

[13] Aberger F., Ruiz I.A.A. (2014). Context-dependent signal integration by the GLI code: The oncogenic load, pathways, modifiers and implications for cancer therapy. Semin. Cell Dev. Biol..

[14] Yang C., Chen W., Chen Y., Jiang J. (2012). Smoothened transduces Hedgehog signal by forming a complex with Evc/Evc2. Cell Res..

[15] Merchant A., Joseph G., Wang Q., Brennan S., Matsui W. (2010). Gli1 regulates the proliferation and differentiation of HSCs and myeloid progenitors. Blood.

[16] Speek M., Njunkova O., Pata I., Valdre E., Kogerman P. (2006). A potential role of alternative splicing in the regulation of the transcriptional activity of human GLI2 in gonadal tissues. BMC Mol. Biol..

[17] Pan Y., Wang B. (2007). A Novel Protein-processing Domain in Gli2 and Gli3 Differentially Blocks Complete Protein Degradation by the Proteasome. J. Biol. Chem..

[18] Palaniswamy R., Teglund S., Lauth M., Zaphiropoulos P.G., Shimokawa T. (2010). Genetic variations regulate alternative splicing in the 5′ untranslated regions of the mouse glioma-associated oncogene 1, Gli1. BMC Mol. Biol..

[19] Shimokawa T., Tostar U., Lauth M., Palaniswamy R., Kasper M., Toftgard R., Zaphiropoulos P.G. (2008). Novel human glioma-associated oncogene 1 (GLI1) splice variants reveal distinct mechanisms in the terminal transduction of the hedgehog signal. J. Biol. Chem..

[20] Cain J.E., Islam E., Haxho F., Blake J., Rosenblum N.D. (2011). GLI3 repressor controls functional development of the mouse ureter. J. Clin. Investig..

[21] Cain J.E., Islam E., Haxho F., Chen L., Bridgewater D., Nieuwenhuis E., Hui C.-C., Rosenblum N.D. (2009). GLI3 repressor controls nephron number via regulation of Wnt11 and Ret in ureteric tip cells. PLoS ONE.

[22] Sheybani-Deloui S., Chi L., Staite M.V., Cain J.E., Nieman B.J., Henkelman R.M., Wainwright B.J., Potter S.S., Bagli D.J., Lorenzo A. (2018). Activated Hedgehog-GLI signaling causes congenital ureteropelvic junction obstruction. J. Am. Soc. Nephrol..

[23] Wen X., Lai C.K., Evangelista M., Hongo J.-A., De Sauvage F.J., Scales S.J. (2010). Kinetics of hedgehog-dependent full-length Gli3 accumulation in primary cilia and subsequent degradation. Mol. Cell. Biol..

[24] Montagnani V., Stecca B. (2019). Role of protein kinases in hedgehog pathway control and implications for cancer therapy. Cancers.

[25] Niewiadomski P., Niedziółka S.M., Markiewicz Ł., Uśpieński T., Baran B., Chojnowska K. (2019). Gli proteins: Regulation in development and cancer. Cells.

[26] Antonucci L., Di Magno L., D’Amico D., Manni S., Serrao S.M., Di Pastena F., Bordone R., Yurtsever Z.N., Caimano M., Petroni M. (2019). Mitogen-activated kinase kinase kinase 1 inhibits hedgehog signaling and medulloblastoma growth through GLI1 phosphorylation. Int. J. Oncol..

[27] Kogerman P., Grimm T., Kogerman L., Krause D., Unden A.B., Sandstedt B., Toftgård R., Zaphiropoulos P.G. (1999). Mammalian suppressor-of-fused modulates nuclear-cytoplasmic shuttling of Gli-1. Nat. Cell Biol..

[28] Han Y., Xiong Y., Shi X., Wu J., Zhao Y., Jiang J. (2017). Regulation of Gli ciliary localization and Hedgehog signaling by the PY-NLS/karyopherin-beta2 nuclear import system. PLoS Biol..

[29] Dishinger J.F., Kee H.L., Jenkins P.M., Fan S., Hurd T.W., Hammond J.W., Truong Y.N., Margolis B., Martens J.R., Verhey K.J. (2010). Ciliary entry of the kinesin-2 motor KIF17 is regulated by importin-beta2 and RanGTP. Nat. Cell Biol..

[30] Niewiadomski P., Kong J.H., Ahrends R., Ma Y., Humke E.W., Khan S., Teruel M.N., Novitch B.G., Rohatgi R. (2014). Gli protein activity is controlled by multisite phosphorylation in vertebrate Hedgehog signaling. Cell Rep..

[31] Pietrobono S., Gagliardi S., Stecca B. (2019). Non-canonical hedgehog signaling pathway in cancer: Activation of GLI transcription factors beyond smoothened. Front. Genet..

[32] Arsic D., Beasley S.W., Sullivan M.J. (2007). Switched-on Sonic hedgehog: A gene whose activity extends beyond fetal development to oncogenesis. J. Paediatr. Child Health.

[33] Cridland S.O., Keys J.R., Papathanasiou P., Perkins A.C. (2009). Indian hedgehog supports definitive erythropoiesis. Blood Cells Mol. Dis..

[34] Bitgood M.J., Shen L., McMahon A.P. (1996). Sertoli cell signaling by Desert hedgehog regulates the male germline. Curr. Biol..

[35] Suzman D.L., Antonarakis E.S. (2015). Clinical implications of hedgehog pathway signaling in prostate cancer. Cancers.

[36] Cohen M., Kicheva A., Ribeiro A., Blassberg R., Page K.M., Barnes C.P., Briscoe J. (2015). Ptch1 and Gli regulate Shh signalling dynamics via multiple mechanisms. Nat. Commun..

[37] Humke E.W., Dorn K.V., Milenkovic L., Scott M.P., Rohatgi R. (2010). The output of Hedgehog signaling is controlled by the dynamic association between Suppressor of Fused and the Gli proteins. Genes Dev..

[38] He M., Subramanian R., Bangs F., Omelchenko T., Jr K.F.L., Kapoor T.M., Anderson K.V. (2014). The kinesin-4 protein Kif7 regulates mammalian Hedgehog signalling by organizing the cilium tip compartment. Nat. Cell Biol..

[39] Chen M.H., Gao N., Kawakami T., Chuang P.T. (2005). Mice deficient in the fused homolog do not exhibit phenotypes indicative of perturbed hedgehog signaling during embryonic development. Mol. Cell Biol..

[40] Stecca B., Ruiz i Altaba A. (2009). A GLI1-p53 inhibitory loop controls neural stem cell and tumour cell numbers. EMBO J..

[41] Pellegrini C., Maturo M.G., Di Nardo L., Ciciarelli V., Gutierrez Garcia-Rodrigo C., Fargnoli M.C. (2017). Understanding the molecular genetics of basal cell carcinoma. Int. J. Mol. Sci..

[42] Di Marcotullio L., Ferretti E., De Smaele E., Argenti B., Mincione C., Zazzeroni F., Gallo R., Masuelli L., Napolitano M., Maroder M. (2004). REN(KCTD11) is a suppressor of Hedgehog signaling and is deleted in human medulloblastoma. Proc. Natl. Acad. Sci. USA.

[43] Stecca B., Mas C., Clement V., Zbinden M., Correa R., Piguet V., Beermann F., i Altaba A.R. (2007). Melanomas require HEDGE-HOG-GLI signaling regulated by interactions between GLI1 and the RAS-MEK/AKT pathways. Proc. Natl. Acad. Sci. USA.

[44] Perrot C.Y., Javelaud D., Mauviel A. (2013). Overlapping activities of TGF-beta and Hedgehog signaling in cancer: Therapeutic targets for cancer treatment. Pharmacol. Ther..

[45] Kern D., Regl G., Hofbauer S.W., Altenhofer P., Achatz G., Dlugosz A., Schnidar H., Greil R., Hartmann T.N., Aberger F. (2015). Hedgehog/GLI and PI3K signaling in the initiation and maintenance of chronic lymphocytic leukemia. Oncogene.

[46] Rajurkar M., De Jesus-Monge W.E., Driscoll D.R., Appleman V.A., Huang H., Cotton J.L., Klimstra D.S., Zhu L.J., Simin K., Xu L. (2012). The activity of Gli transcription factors is essential for Kras-induced pancreatic tumorigenesis. Proc. Natl. Acad. Sci. USA.

[47] Ram Kumar R.M., Betz M.M., Robl B., Born W., Fuchs B. (2014). DeltaNp63alpha enhances the oncogenic phenotype of osteosarcoma cells by inducing the expression of GLI2. BMC Cancer.

[48] Cochrane C.R., Szczepny A., Watkins D.N., Cain J.E. (2015). Hedgehog signaling in the maintenance of cancer stem cells. Cancers.

[49] Brandner S. (2010). Nanog, Gli, and p53: A new network of stemness in development and cancer. EMBO J..

[50] Bora-Singhal N., Perumal D., Nguyen J., Chellappan S.P. (2015). Gli1-mediated regulation of Sox2 facilitates self-renewal of stem-like cells and confers resistance to EGFR inhibitors in non–small cell lung cancer. Neoplasia.

[51] Gonnissen A., Isebaert S., Haustermans K. (2015). Targeting the Hedgehog signaling pathway in cancer: Beyond Smoothened. Oncotarget.

[52] Wang Y., Ding Q., Yen C.-J., Xia W., Izzo J.G., Lang J.-Y., Li C.-W., Hsu J.L., Miller S.A., Wang X. (2012). The Crosstalk of mTOR/S6K1 and hedgehog pathways. Cancer Cell.

[53] Nandagopal N., Santat L.A., LeBon L., Sprinzak D., Bronner M.E., Elowitz M.B. (2018). Dynamic ligand discrimination in the notch signaling pathway. Cell.

[54] Palle K., Mani C., Tripathi K., Athar M. (2015). Aberrant GLI1 Activation in DNA Damage Response, Carcinogenesis and Chemoresistance. Cancers.

[55] Gu D., Xie J. (2015). Non-Canonical Hh Signaling in Cancer—Current Understanding and Future Directions. Cancers.

[56] Porter J.A., Von Kessler D.P., Ekker S.C., Young K.E., Lee J.J., Moses K., Beachy P.A. (1995). The product of hedgehog autoproteolytic cleavage active in local and long-range signalling. Nat. Cell Biol..

[57] Jenkins D. (2009). Hedgehog signalling: Emerging evidence for non-canonical pathways. Cell. Signal..

[58] Elia D., Madhala D., Ardon E., Reshef R., Halevy O. (2007). Sonic hedgehog promotes proliferation and differentiation of adult muscle cells: Involvement of MAPK/ERK and PI3K/Akt pathways. Biochim. Bio-Phys. Acta.

[59] Mangelberger D., Kern D., Loipetzberger A., Eberl M., Aberger F. (2012). Cooperative Hedgehog-EGFR signaling. Front. Biosci..

[60] Medina V., Calvo M.B., Díaz-Prado S., Espada J. (2009). Hedgehog signalling as a target in cancer stem cells. Clin. Transl. Oncol..

[61] Carpenter R.L., Lo H.-W. (2012). Hedgehog pathway and GLI1 isoforms in human cancer. Discov. Med..

[62] Li L., Tao Y., Mao J., Zhang Q. (2011). Overexpression of Hedgehog signaling molecules and its involvement in triple-negative breast cancer. Oncol. Lett..

[63] Noman A.S., Uddin M.T., Rahman M.Z., Nayeem M.J., Alam S.S., Khatun Z., Wahiduzzaman M., Sultana A., Ali M.Y., Barua D. (2016). Overexpression of sonic hedgehog in the triple negative breast cancer: Clinicopathological characteristics of high burden breast cancer patients from Bangladesh. Sci. Rep..

[64] Islam S., Mokhtari R., Noman A., Uddin M., Rahman M., Azadi M., Zlotta A., Van Der Kwast T., Yeger H., Farhat W. (2016). Sonic hedgehog (Shh) signaling promotes tumorigenicity and stemness via activation of epithelial-to-mesenchymal transition (EMT) in bladder cancer. Mol. Carcinog..

[65] Wang L., Choi Y.L., Hua X.Y., Shin Y.K., Song Y.J., Youn S.J., Yun H.Y., Park S.M., Kim W.J., Kim H.J. (2006). Increased expression of sonic hedgehog and altered methylation of its promoter region in gastric cancer and its related lesions. Mod. Pathol..

[66] Szkandera J., Kiesslich T., Haybaeck J., Gerger A., Pichler M. (2013). Hedgehog signaling pathway in ovarian cancer. Int. J. Mol. Sci..

[67] Wang B., Yu T., Hu Y., Xiang M., Peng H., Lin Y., Han L., Zhang L. (2017). Prognostic role of Gli1 expression in breast cancer: A meta-analysis. Oncotarget.

[68] Han B., Qu Y., Yu-Rice Y., Johnson J., Cui X. (2016). FOXC1-induced Gli2 activation: A non-canonical pathway contributing to stemness and anti-Hedgehog resistance in basal-like breast cancer. Mol. Cell. Oncol..

[69] Han B., Qu Y., Jin Y., Yu Y., Deng N., Wawrowsky K., Zhang X., Li N., Bose S., Wang Q. (2015). FOXC1 activates smoothened-independent hedgehog signaling in basal-like breast cancer. Cell Rep..

[70] Di Mauro C., Rosa R., D’Amato V., Ciciola P., Servetto A., Marciano R., Orsini R.C., Formisano L., De Falco S., Cicatiello V. (2017). Hedgehog signalling pathway orchestrates angiogenesis in triple-negative breast cancers. Br. J. Cancer.

[71] Thomas Z., Gibson W.T., Sexton J.Z., Aird K.M., Ingram S.M., Aldrich A.J., Lyerly H.K., Devi G.R., Williams K.P. (2011). Targeting GLI1 expression in human inflammatory breast cancer cells enhances apoptosis and attenuates migration. Br. J. Cancer.

[72] Riaz S.K., Khan J.S., Shah S.T.A., Wang F., Ye L., Jiang W.G., Malik M.F.A. (2018). Involvement of hedgehog pathway in early onset, aggressive molecular subtypes and metastatic potential of breast cancer. Cell Commun. Signal..

[73] Kasper M., Jaks V., Fiaschi M., Toftgård R. (2009). Hedgehog signalling in breast cancer. Carcinogenesis.

[74] Sari I.N., Phi L.T.H., Jun N., Wijaya Y.T., Lee S., Kwon H.Y. (2018). Hedgehog signaling in cancer: A prospective therapeutic target for eradicating cancer stem cells. Cells.

[75] Song Y., Zhang J., Tian T., Fu X., Wang W., Li S., Shi T., Suo A., Ruan Z., Guo H. (2016). SET7/9 inhibits oncogenic activities through regulation of Gli-1 expression in breast cancer. Tumour Biol..

[76] Colavito S.A., Zou M.R., Yan Q., Nguyen D.X., Stern D.F. (2014). Significance of glioma-associated oncogene homolog 1 (GLI1) expression in claudin-low breast cancer and crosstalk with the nuclear factor kappa-light-chain-enhancer of activated B cells (NF-κB) pathway. Breast Cancer Res..

[77] Anderson W.F., Schairer C., Chen B.E., Hance K.W., Levine P.H. (2006). Epidemiology of inflammatory breast cancer (IBC)1. Breast Dis..

[78] Hance K.W., Anderson W.F., Devesa S.S., Young H.A., Levine P.H. (2005). Trends in inflammatory breast carcinoma incidence and survival: The surveillance, epidemiology, and end results program at the National Cancer Institute. J. Natl. Cancer Inst..

[79] Denis G.V., Sebastiani P., Bertrand K.A., Strissel K.J., Tran A.H., Slama J., Medina N.D., Andrieu G., Palmer J.R. (2018). Inflammatory signatures distinguish metabolic health in African American women with obesity. PLoS ONE.

[80] Habib J.G., O’Shaughnessy J.A. (2016). The hedgehog pathway in triple-negative breast cancer. Cancer Med..

[81] Carpenter R.L., Lo H.W. (2012). Identification, functional characterization, and pathobiological significance of GLI1 isoforms in human cancers. Vitam. Horm..

[82] Han Y.-G., Kim H.J., Dlugosz A.A., Ellison D.W., Gilbertson R.J., Alvarez-Buylla A. (2009). Dual and opposing roles of primary cilia in medulloblastoma development. Nat. Med..

[83] Koso H., Tsuhako A., Lyons E., Ward J.M., Rust A.G., Adams D.J., Jenkins N.A., Copeland N.G., Watanabe S. (2014). Identification of FoxR2 as an oncogene in medulloblastoma. Cancer Res..

[84] Milla L.A., Arros A., Espinoza N., Remke M., Kool M., Taylor M.D., Pfister S.M., Wainwright B., Palma V. (2013). Neogenin1 is a sonic hedgehog target in medulloblastoma and is necessary for cell cycle progression. Int. J. Cancer.

[85] Rajurkar M., Huang H., Cotton J.L., Brooks J.K., Sicklick J., McMahon A.P., Mao J. (2013). Distinct cellular origin and genetic requirement of Hedgehog-Gli in postnatal rhabdomyosarcoma genesis. Oncogene.

[86] Jagani Z., Mora-Blanco E.L., Sansam C.G., McKenna E.S., Wilson B., Chen D., Klekota J., Tamayo P., Nguyen P.T.L., Tolstorukov M. (2010). Loss of the tumor suppressor Snf5 leads to aberrant activation of the Hedgehog-Gli pathway. Nat. Med..

[87] Fu J., Rodova M., Roy S.K., Sharma J., Singh K.P., Srivastava R.K., Shankar S. (2013). GANT-61 inhibits pancreatic cancer stem cell growth in vitro and in NOD/SCID/IL2R gamma null mice xenograft. Cancer Lett..

[88] Miyazaki Y., Matsubara S., Ding Q., Tsukasa K., Yoshimitsu M., Kosai K.I., Takao S. (2016). Efficient elimination of pancreatic cancer stem cells by hedgehog/GLI inhibitor GANT61 in combination with mTOR inhibition. Mol. Cancer.

[89] Chiang C., Litingtung Y., Lee E., Young K.E., Corden J.L., Westphal H., Beachy P.A. (1996). Cyclopia and defective axial patterning in mice lacking Sonic hedgehog gene function. Nat. Cell Biol..

[90] Freestone S.H., Marker P., Grace O., Tomlinson D.C., Cunha G.R., Harnden P., Thomson A.A. (2003). Sonic hedgehog regulates prostatic growth and epithelial differentiation. Dev. Biol..

[91] Bhandari A., Woodhouse M., Gupta S. (2017). Colorectal cancer is a leading cause of cancer incidence and mortality among adults younger than 50 years in the USA: A SEER-based analysis with comparison to other young-onset cancers. J. Investig. Med..

[92] Singovski G., Bernal C., Kuciak M., Siegl-Cachedenier I., Conod A., Ruiz I.A.A. (2016). In vivo epigenetic reprogramming of primary human colon cancer cells enhances metastases. J. Mol. Cell Biol..

[93] Regan J.L. (2018). Cell fate in colon cancer stem cells: To GLI or not to GLI?. Mol. Cell. Oncol..

[94] Regan J.L., Schumacher D., Staudte S., Steffen A., Haybaeck J., Keilholz U., Schweiger C., Golob-Schwarzl N., Mumberg D., Henderson D. (2017). Non-canonical hedgehog signaling is a positive regulator of the WNT pathway and is required for the survival of colon cancer stem cells. Cell Rep..

[95] Di Magno L., Coni S., Di Marcotullio L., Canettieri G. (2015). Digging a hole under Hedgehog: Downstream inhibition as an emerging anticancer strategy. Biochim. Biophys. Acta.

[96] Mazumdar T., DeVecchio J., Agyeman A., Shi T., Houghton J.A. (2011). The GLI genes as the molecular switch in disrupting Hedgehog signaling in colon cancer. Oncotarget.

[97] Yang Q., Shen S.S., Zhou S., Ni J., Chen D., Wang G., Yuan L. (2012). STAT3 activation and aberrant ligand-dependent sonic hedgehog signaling in human pulmonary adenocarcinoma. Exp. Mol. Pathol..

[98] Rizvi S., Demars C.J., Comba A., Gainullin V.G., Rizvi Z., Almada L.L., Wang K., Lomberk G., Fernández-Zapico M.E., Buttar N.S. (2010). Combinatorial chemoprevention reveals a novel smoothened-independent role of GLI1 in esophageal carcinogenesis. Cancer Res..

[99] Yang L., Wang L.S., Chen X.L., Gatalica Z., Qiu S., Liu Z., Stoner G., Zhang H., Weiss H., Xie J. (2012). Hedgehog signaling activation in the de-velopment of squamous cell carcinoma and adenocarcinoma of esophagus. Int. J. Biochem. Mol. Biol..

[100] Merchant J.L., Ding L. (2017). Hedgehog signaling links chronic inflammation to gastric cancer precursor lesions. Cell. Mol. Gastroenterol. Hepatol..

[101] El-Zaatari M., Kao J.Y., Tessier A., Bai L., Hayes M.M., Fontaine C., Eaton K.A., Merchant J.L. (2013). Gli1 deletion prevents Helicobacter-induced gastric metaplasia and expansion of myeloid cell subsets. PLoS ONE.

[102] Zeng C., Wang Y., Lu Q., Chen J., Zhang J., Liu T., Lv N., Luo S. (2014). SPOP suppresses tumorigenesis by regulating Hedgehog/Gli2 signaling pathway in gastric cancer. J. Exp. Clin. Cancer Res..

[103] Lee S.J., Do I.G., Lee J., Kim K., Jang J., Sohn I., Kang W.K. (2013). Gastric cancer (GC) patients with hedgehog pathway activation: PTCH1 and GLI2 as independent prognostic factors. Target. Oncol..

[104] Wong S.Y., Seol A.D., So P.-L., Ermilov A.N., Bichakjian C.K., Epstein E.H., Dlugosz A.A., Reiter J.F. (2009). Primary cilia can both mediate and suppress Hedgehog pathway–dependent tumorigenesis. Nat. Med..

[105] Pandolfi S., Stecca B. (2015). Cooperative integration between HEDGEHOG-GLI signalling and other oncogenic pathways: Implications for cancer therapy. Expert Rev. Mol. Med..

[106] Grachtchouk M., Mo R., Yu S., Zhang X., Sasaki H., Hui C.C., Dlugosz A.A. (2000). Basal cell carcinomas in mice over-expressing Gli2 in skin. Nat. Genet..

[107] Fei D.L., Sanchez-Mejias A., Wang Z., Flaveny C., Long J., Singh S., Rodriguez-Blanco J., Tokhunts R., Giambelli C., Briegel K.J. (2012). Hedgehog signaling regulates bladder cancer growth and tumorigenicity. Cancer Res..

[108] Ok C.Y., Singh R.R., Vega F. (2012). Aberrant activation of the hedgehog signaling pathway in malignant hematological neoplasms. Am. J. Pathol..

[109] Singh R.R., Kim J.E., Davuluri Y., Drakos E., Cho-Vega J.H., Amin H.M., Vega F. (2010). Hedgehog signaling pathway is activated in diffuse large B-cell lymphoma and contributes to tumor cell survival and proliferation. Leukemia.

[110] Jagani Z., Dorsch M., Warmuth M. (2010). Hedgehog pathway activation in chronic myeloid leukemia: A promise for a novel combination therapeutic approach?. Cell Cycle.

[111] Blotta S., Jakubikova J., Calimeri T., Roccaro A.M., Amodio N., Azab A.K., Foresta U., Mitsiades C.S., Rossi M., Todoerti K. (2012). Canonical and non-canonical Hedgehog pathway in the pathogenesis of multiple myeloma. Blood.

[112] Perez-Galan P., Dreyling M., Wiestner A. (2011). Mantle cell lymphoma: Biology, pathogenesis, and the molecular basis of treatment in the genomic era. Blood.

[113] Hegde G.V., Munger C.M., Emanuel K., Joshi A.D., Greiner T.C., Weisenburger D.D., Vose J.M., Joshi S.S. (2008). Targeting of sonic hedgehog-GLI signaling: A potential strategy to improve therapy for mantle cell lymphoma. Mol. Cancer Ther..

[114] Greaves W.O., Kim J.E., Singh R.R., Drakos E., Kunkalla K., Sánchez-Espiridión B., Garcia J.F., Medeiros L.J., Vega F. (2011). Glioma-associated oncogene homologue 3, a hedgehog transcription factor, is highly expressed in Hodgkin and Reed-Sternberg cells of classical Hodgkin lymphoma. Hum. Pathol..

[115] Yuan Z., Goetz J.A., Singh S., Ogden S.K., Petty W.J., Black C.C., Memoli V.A., Dmitrovsky E., Robbins D.J. (2006). Frequent requirement of hedgehog signaling in non-small cell lung carcinoma. Oncogene.

[116] Rubin J.B., Rowitch D.H. (2002). Medulloblastoma: A problem of developmental biology. Cancer Cell.

[117] Taylor M.D., Liu L., Raffel C., Hui C.-C., Mainprize T.G., Zhang X., Agatep R., Chiappa S., Gao L., Lowrance A. (2002). Mutations in SUFU predispose to medulloblastoma. Nat. Genet..

[118] Athar M., Li C., Kim A.L., Spiegelman V.S., Bickers D.R. (2014). Sonic hedgehog signaling in basal cell nevus syndrome. Cancer Res..

[119] Richieri-Costa A., Vendramini-Pittoli S., Kokitsu-Nakata N.M., Zechi-Ceide R.M., Alvarez C.W., Ribei-ro-Bicudo L.A. (2017). Multisystem involvement in a patient with a PTCH1 mutation: Clinical and imaging findings. J. Pediatr. Genet..

[120] Xie J., Murone M., Luoh S.M., Ryan A., Gu Q., Zhang C., Bonifas J.M., Lam C.W., Hynes M., Goddard A. (1998). Activating smoothened mutations in sporadic basal-cell carcinoma. Nature.

[121] Macdonald T.J. (2012). Hedgehog pathway in pediatric cancers: They’re not just for brain tumors anymore. Am. Soc. Clin. Oncol. Educ. Book.

[122] Carpenter R.L., Paw I., Zhu H., Sirkisoon S., Xing F., Watabe K., Debinski W., Lo H. (2015). The gain-of-function GLI1 transcription factor TGLI1 enhances expression of VEGF-C and TEM7 to promote glioblastoma angiogenesis. Oncotarget.

[123] Babic A.M., Kireeva M.L., Kolesnikova T.V., Lau L.F. (1998). CYR61, a product of a growth factor-inducible immediate early gene, promotes angiogenesis and tumor growth. Proc. Natl. Acad. Sci. USA.

[124] Chen C.C., Mo F.E., Lau L.F. (2001). The angiogenic factor Cyr61 activates a genetic program for wound healing in human skin fibroblasts. J. Biol. Chem..

[125] Harris L.G., Pannell L.K., Singh S., Samant R.S., Shevde L.A. (2012). Increased vascularity and spontaneous metastasis of breast cancer by hedgehog signaling mediated upregulation of cyr61. Oncogene.

[126] Renault M.-A., Roncalli J., Tongers J., Thorne T., Klyachko E., Misener S., Volpert O.V., Mehta S., Burg A., Luedemann C. (2010). Sonic hedgehog induces angiogenesis via Rho kinase-dependent signaling in endothelial cells. J. Mol. Cell. Cardiol..

[127] Teng H., Chopp M., Hozeska-Solgot A., Shen L., Lü M., Tang C., Zhang Z.G. (2012). Tissue plasminogen activator and plasminogen activator inhibitor 1 contribute to sonic hedgehog-induced in vitro cerebral angiogenesis. PLoS ONE.

[128] Sun M., Zhang N., Wang X., Li Y., Qi W., Zhang H., Li Z., Yang Q. (2016). Hedgehog pathway is involved in nitidine chloride induced inhibition of epithelial-mesenchymal transition and cancer stem cells-like properties in breast cancer cells. Cell Biosci..

[129] Mani S.A., Guo W., Liao M.J., Eaton E.N., Ayyanan A., Zhou A.Y., Brooks M., Reinhard F., Zhang C.C., Shipitsin M. (2008). The epithelial-mesenchymal transition generates cells with properties of stem cells. Cell.

[130] Hay E.D., Zuk A. (1995). Transformations between epithelium and mesenchyme: Normal, pathological, and experimentally induced. Am. J. Kidney Dis..

[131] Hay E.D. (1995). An Overview of epithelio-mesenchymal transformation. Cells Tissues Organs.

[132] Tang C., Ximei W., Pan L., Xiong W., Zhu H., Ruan H., Zou C., Tang L., Iguchi T., Wu X.K. (2015). Hedgehog signaling through GLI1 and GLI2 is required for epithelial–mesenchymal transition in human trophoblasts. Biochim. Biophys. Acta.

[133] Park J.W., Park D.M., Choi B.K., Kwon B.S., Seong J.K., Green J.E., Kim D.-Y., Kim H.K. (2014). Establishment and characterization of metastatic gastric cancer cell lines from murine gastric adenocarcinoma lacking Smad4, p53, and E-cadherin. Mol. Carcinog..

[134] Kim K., Daniels K.J., Hay E.D. (1998). Tissue-specific expression of beta-catenin in normal mesenchyme and uveal melanomas and its effect on invasiveness. Exp. Cell Res..

[135] Liu Y., Zeng C., Bao N., Zhao J., Hu Y., Li C., Chi S. (2015). Effect of Rab23 on the proliferation and apoptosis in breast cancer. Oncol. Rep..

[136] Chi S., Xie G., Liu H., Chen K., Zhang X., Li C., Xie J. (2012). Rab23 negatively regulates Gli1 transcriptional factor in a Su(Fu)-dependent manner. Cell. Signal..

[137] Eggenschwiler J.T., Bulgakov O.V., Qin J., Li T., Anderson K.V. (2006). Mouse Rab23 regulates hedgehog signaling from smoothened to Gli proteins. Dev. Biol..

[138] Neelakantan D., Zhou H., Oliphant M.U.J., Zhang X., Simon L.M., Henke D.M., Shaw C.A., Wu M.-F., Hilsenbeck S.G., White L.D. (2017). EMT cells increase breast cancer metastasis via paracrine GLI activation in neighbouring tumour cells. Nat. Commun..

[139] Chaudhry P., Singh M., Triche T.J., Guzman M., Merchant A.A. (2017). GLI3 repressor determines Hedgehog pathway activation and is required for response to SMO antagonist glasdegib in AML. Blood.

[140] Lopez-Rios J., Speziale D., Robay D., Scotti M., Osterwalder M., Nusspaumer G., Galli A., Holländer G.A., Kmita M., Zeller R. (2012). GLI3 constrains digit number by controlling both progenitor proliferation and BMP-dependent exit to chondrogenesis. Dev. Cell.

[141] Kaplan D.H., Shankaran V., Dighe A.S., Stockert E., Aguet M., Old L.J., Schreiber R.D. (1998). Demonstration of an interferon gamma-dependent tumor surveillance system in immunocompetent mice. Proc. Natl. Acad. Sci. USA.

[142] Laner-Plamberger S., Wolff F., Kaser-Eichberger A., Swierczynski S., Hauser-Kronberger C., Frischauf A.-M., Eichberger T. (2013). Hedgehog/GLI signaling activates suppressor of cytokine signaling 1 (SOCS1) in epidermal and neural tumor cells. PLoS ONE.

[143] Velcheti V., Govindan R. (2007). Hedgehog signaling pathway and lung cancer. J. Thorac. Oncol..

[144] Fan H., Khavari P.A., Blagoveshchenskaya A.D., Hewitt E.W., Cutler D.F. (1999). Sonic hedgehog opposes epithelial cell cycle arrest. J. Cell Biol..

[145] Inaguma S., Ito H., Riku M., Ikeda H., Kasai K. (2015). Addiction of pancreatic cancer cells to zinc-finger transcription factor ZIC2. Oncotarget.

[146] Singh A.K., Arya R.K., Trivedi A.K., Sanyal S., Baral R., Dormond O., Briscoe D.M., Datta D. (2013). Chemokine receptor trio: CXCR3, CXCR4 and CXCR7 crosstalk via CXCL11 and CXCL12. Cytokine Growth Factor Rev..

[147] Inaguma S., Riku M., Ito H., Tsunoda T., Ikeda H., Kasai K. (2015). GLI1 orchestrates CXCR4/CXCR7 signaling to enhance migration and metastasis of breast cancer cells. Oncotarget.

[148] Pizarro A., Benito N., Navarro P., Palacios J., Cano A., Quintanilla M., Contreras F., Gamallo C. (1994). E-cadherin expression in basal cell carcinoma. Br. J. Cancer.

[149] Koorstra J.B., Karikari C.A., Feldmann G., Bisht S., Rojas P.L., Offerhaus G.J., Alvarez H., Maitra A. (2009). The Axl receptor tyro-sine kinase confers an adverse prognostic influence in pancreatic cancer and represents a new therapeutic target. Cancer Biol. Ther..

[150] Kim Y., Yoon J.W., Xiao X., Dean N.M., Monia B.P., Marcusson E.G. (2007). Selective down-regulation of glioma-associated oncogene 2 inhibits the proliferation of hepatocellular carcinoma cells. Cancer Res..

[151] Kim Y.S., Kang H.S., Jetten A.M. (2007). The Kruppel-like zinc finger protein Glis2 functions as a negative modulator of the Wnt/beta-catenin signaling pathway. FEBS Lett..

[152] Dakhova O., Rowley D., Ittmann M. (2014). Genes upregulated in prostate cancer reactive stroma promote prostate cancer progression in vivo. Clin. Cancer Res..

[153] Cunha G., Hayward S.W., Dahiya R., Foster B. (1996). Smooth muscle-epithelial interactions in normal and neoplastic prostatic development. Cells Tissues Organs.

[154] Yauch R.L., Gould S.E., Scales S.J., Tang T., Tian H., Ahn C.P., Marshall D., Fu L., Januario T., Kallop D. (2008). A paracrine requirement for hedgehog signalling in cancer. Nat. Cell Biol..

[155] Fan L., Pepicelli C.V., Dibble C.C., Catbagan W., Zarycki J.L., Laciak R., Gipp J., Shaw A., Lamm M.L.G., Munoz A. (2004). Hedgehog signaling promotes prostate xenograft tumor growth. Endocrinology.

[156] Tzelepi V., Karlou M., Wen S., Hoang A., Logothetis C., Troncoso P., Efstathiou E. (2011). Expression of hedgehog pathway components in prostate carcinoma microenvironment: Shifting the balance towards autocrine signalling. Histopathology.

[157] Smelkinson M.G. (2017). The Hedgehog signaling pathway emerges as a pathogenic target. J. Dev. Biol..

[158] Sabol M., Trnski D., Musani V., Ozretić P., Levanat S. (2018). Role of GLI transcription factors in pathogenesis and their potential as new therapeutic targets. Int. J. Mol. Sci..

[159] Liu D., Zeinolabediny Y., Caccuri F., Ferris G., Fang W.-H., Weston R., Krupinski J., Colombo L., Salmona M., Corpas R. (2019). p17 from HIV induces brain endothelial cell angiogenesis through EGFR-1-mediated cell signalling activation. Lab. Investig..

[160] Caccuri F., Giagulli C., Bugatti A., Benetti A., Alessandri G., Ribatti D., Marsico S., Apostoli P., Slevin M.A., Rusnati M. (2012). HIV-1 matrix protein p17 promotes angiogenesis via chemokine receptors CXCR1 and CXCR2. Proc. Natl. Acad. Sci. USA.

[161] Benkheil M., Paeshuyse J., Neyts J., Van Haele M., Roskams T., Liekens S. (2018). HCV-induced EGFR-ERK signaling promotes a pro-inflammatory and pro-angiogenic signature contributing to liver cancer patho-genesis. Biochem. Pharmacol..

[162] Alkharsah K.R. (2018). VEGF upregulation in viral infections and its possible therapeutic implications. Int. J. Mol. Sci..

[163] Vrancken K., Paeshuyse J., Liekens S. (2012). Angiogenic activity of hepatitis B and C viruses. Antivir. Chem. Chemother..

[164] Paydas S., Ergin M., Erdogan S., Seydaoglu G. (2008). Prognostic significance of EBV-LMP1 and VEGF-A expressions in non-Hodgkin’s lymphomas. Leuk. Res..

[165] Sharma-Walia N., Paul A.G., Bottero V., Sadagopan S., Veettil M.V., Kerur N., Chandran B. (2010). Kaposi’s sarcoma associated herpes virus (KSHV) induced COX-2: A key factor in latency, inflammation, angiogenesis, cell survival and invasion. PLoS Pathog..

[166] Rivera-Soto R., Damania B. (2019). Modulation of angiogenic processes by the human gammaherpesviruses, epstein–barr virus and kaposi’s sarcoma-associated herpesvirus. Front. Microbiol..

[167] Coultas L., Nieuwenhuis E., Anderson G.A., Cabezas J., Nagy A., Henkelman R.M., Hui C.C., Rossant J. (2010). Hedgehog regu-lates distinct vascular patterning events through VEGF-dependent and -independent mechanisms. Blood.

[168] Li Y., Xia Y., Wang Y., Mao L., Gao Y., He Q., Huang M., Chen S., Hu B. (2013). Sonic hedgehog (Shh) regulates the expression of angiogenic growth factors in oxygen–glucose-deprived astrocytes by mediating the nuclear receptor NR2F2. Mol. Neurobiol..

[169] Mbhele N., Moodley J., Naicker T. (2017). Role of angiopoietin-2, endoglin, and placental growth factor in HIV-associated preeclampsia. Hypertens. Pregnancy.

[170] Foka P., Karamichali E., Dalagiorgou G., Serti E., Doumba P.P., Pissas G., Kakkanas A., Kazazi D., Kochlios E., Gaitanou M. (2014). Hepatitis C virus modu-lates lipid regulatory factor Angiopoietin-like 3 gene expression by repressing HNF-1alpha activity. J. Hepatol..

[171] Li Y., Chen J., Wu C., Wang L., Lu M., Chen X. (2010). Hepatitis B virus/hepatitis C virus upregulate angiopoi-etin-2 expression through mitogen-activated protein kinase pathway. Hepatol. Res..

[172] Sanz-Cameno P., Martin-Vilchez S., Lara-Pezzi E., Borque M.J., Salmeron J., Munoz de Rueda P., Solís J.A., López-Cabrera M., Moreno-Otero R. (2006). Hepatitis B virus promotes angiopoietin-2 expression in liver tissue: Role of HBV x protein. Am. J. Pathol..

[173] Paudel N., Sadagopan S., Chakraborty S., Sarek G., Ojala P.M., Chandran B. (2012). Kaposi’s sarcoma-associated herpesvirus latency-associated nuclear antigen interacts with multifunctional angiogenin to utilize its antiapoptotic functions. J. Virol..

[174] Ueda K., Ito E., Karayama M., Ohsaki E., Nakano K., Watanabe S. (2010). KSHV-infected PEL cell lines exhibit a distinct gene expression profile. Biochem. Biophys. Res. Commun..

[175] Ma T., Jham B.C., Hu J., Friedman E.R., Basile J.R., Molinolo A., Sodhi A., Montaner S. (2010). Viral G protein-coupled receptor up-regulates Angiopoietin-like 4 promoting angiogenesis and vascular permeability in Kaposi’s sarcoma. Proc. Natl. Acad. Sci. USA.

[176] Yu X., Sha J., Xiang S., Qin S., Conrad P., Ghosh S.K., Weinberg A., Ye F. (2016). Suppression of KSHV-induced angiopoietin-2 inhibits angiogenesis, infiltration of inflammatory cells, and tumor growth. Cell Cycle.

[177] Yao Q., Renault M.-A., Chapouly C., Vandierdonck S., Belloc I., Jaspard-Vinassa B., Daniel-Lamazière J.-M., Laffargue M., Merched A., Desgranges C. (2014). Sonic hedgehog mediates a novel pathway of PDGF-BB–dependent vessel maturation. Blood.

[178] Cavallin L.E., Ma Q., Naipauer J., Gupta S., Kurian M., Locatelli P., Romanelli P., Nadji M., Goldschmidt-Clermont P.J., Mesri E.A. (2018). KSHV-induced ligand mediated activation of PDGF receptor-alpha drives Kaposi’s sarcomagenesis. PLoS Pathog..

[179] Wang G., Zhang Z., Xu Z., Yin H., Bai L., Ma Z., DeCoster M.A., Qian G.-S., Wu G. (2010). Activation of the sonic hedgehog signaling controls human pulmonary arterial smooth muscle cell proliferation in response to hypoxia. Biochim. Biophys. Acta.

[180] Sharma S., Wang J., Alqassim E., Portwood S., Abrams S.I., Maguire O., Basse P.H., Wang E.S., Segal B., Baysal B.E. (2019). Mitochondrial hypoxic stress induces widespread RNA editing by APOBEC3G in natural killer cells. Genome Biol..

[181] Wakisaka N., Kondo S., Yoshizaki T., Murono S., Furukawa M., Pagano J.S. (2004). Epstein-barr virus latent membrane protein 1 induces synthesis of hypoxia-inducible factor 1α. Mol. Cell. Biol..

[182] Cuninghame S., Jackson R., Zehbe I. (2014). Hypoxia-inducible factor 1 and its role in viral carcinogenesis. Virology.

[183] Shin Y.C., Joo C.H., Gack M.U., Lee H.R., Jung J.U. (2008). Kaposi’s sarcoma-associated herpesvirus viral IFN regulatory factor 3 stabilizes hypoxia-inducible factor-1 alpha to induce vascular endothelial growth factor expression. Cancer Res..

[184] Shrestha P., Davis D.A., Veeranna R.P., Carey R.F., Viollet C., Yarchoan R. (2017). Hypoxia-inducible factor-1 alpha as a therapeutic target for primary effusion lymphoma. PLoS Pathog..

[185] Cai Q., Murakami M., Si H., Robertson E.S. (2007). A potential alpha-helix motif in the amino terminus of LANA encoded by Kaposi’s sarcoma-associated herpesvirus is critical for nuclear accumulation of HIF-1alpha in normoxia. J. Virol..

[186] Carroll P.A., Kenerson H.L., Yeung R.S., Lagunoff M. (2006). Latent Kaposi’s sarcoma-associated herpesvirus infection of endothelial cells activates hypoxia-induced factors. J. Virol..

[187] Abe M., Koga H., Yoshida T., Masuda H., Iwamoto H., Sakata M., Hanada S., Nakamura T., Taniguchi E., Kawaguchi T. (2012). Hepatitis C virus core protein upregulates the expression of vascular endothelial growth factor via the nuclear factor-kappaB/hypoxia-inducible factor-1 alpha axis under hypoxic conditions. Hepatol Res..

[188] Klusza S., Deng W.-M. (2010). At the crossroads of differentiation and proliferation: Precise control of cell-cycle changes by multiple signaling pathways in Drosophila follicle cells. BioEssays.

[189] Jiang J., Hui C.-C. (2008). Hedgehog signaling in development and cancer. Dev. Cell.

[190] Lee R.T.H., Zhao Z., Ingham P.W. (2016). Hedgehog signalling. Development.

[191] Smelkinson M.G., Guichard A., Teijaro J.R., Malur M., Loureiro M.E., Jain P., Ganesan S., Zúñiga E.I., Krug R.M., Oldstone M.B. (2017). Influenza NS1 directly modulates Hedgehog signaling during infection. PLoS Pathog..

[192] Sui B., Bamba D., Weng K., Ung H., Chang S., Van Dyke J., Goldblatt M., Duan R., Kinch M.S., Li W.-B. (2009). The use of Random Homozygous Gene Perturbation to identify novel host-oriented targets for influenza. Virology.

[193] Chuang P.T., Kawcak T., McMahon A.P. (2003). Feedback control of mammalian Hedgehog signaling by the Hedgehog-binding protein, Hip1, modulates Fgf signaling during branching morphogenesis of the lung. Genes Dev..

[194] Warburton D., Bellusci S., De Langhe S., Del Moral P.-M., Fleury V., Mailleux A., Tefft D., Unbekandt M., Wang K., Shi W. (2005). Molecular mechanisms of early lung specification and branching morphogenesis. Pediatr. Res..

[195] Giroux-Leprieur E., Costantini A., Ding V.W., He B. (2018). Hedgehog signaling in lung cancer: From oncogenesis to cancer treatment resistance. Int. J. Mol. Sci..

[196] Stewart G.A., Hoyne G.F., Ahmad S.A., Jarman E., Wallace W.A.H., Harrison D.J., Haslett C., Lamb J.R., Howie S.E.M. (2003). Expression of the developmental Sonic hedgehog (Shh) signalling pathway is up-regulated in chronic lung fibrosis and the Shh receptor patched 1 is present in circulating T lymphocytes. J. Pathol..

[197] Piekna-Przybylska D., Sharma G., Maggirwar S.B., Bambara R.A. (2017). Deficiency in DNA damage response, a new characteristic of cells infected with latent HIV-1. Cell Cycle.

[198] Ariumi Y., Kuroki M., Dansako H., Abe K., Ikeda M., Wakita T., Kato N. (2008). The DNA damage sensors ataxia-telangiectasia mutated kinase and checkpoint kinase 2 are required for hepatitis C virus RNA replication. J. Virol..

[199] Wang W.H., Hullinger R.L., Andrisani O.M. (2008). Hepatitis B virus X protein via the p38MAPK pathway induces E2F1 release and ATR kinase activation mediating p53 apoptosis. J. Biol. Chem..

[200] Tatfi M., Hermine O., Suarez F. (2019). Epstein-barr virus (EBV)-related lymphoproliferative disorders in ataxia telangiectasia: Does ATM regulate EBV life cycle?. Front. Immunol..

[201] Uppal T., Sarkar R., Dhelaria R., Verma S.C. (2018). Role of pattern recognition receptors in KSHV infection. Cancers.

[202] Kumar A., Sahu S.K., Mohanty S., Chakrabarti S., Maji S., Reddy R.R., Jha A., Goswami C., Kundu C., Rajasubramaniam S. (2014). Kaposi sarcoma herpes virus latency associated nuclear antigen protein release the G2/M cell cycle blocks by modulating ATM/ATR mediated checkpoint pathway. PLoS ONE.

[203] Singh V.V., Dutta D., Ansari M.A., Dutta S., Chandran B. (2014). Kaposi’s sarcoma-associated herpesvirus induces the ATM and H2AX DNA damage response early during de novo infection of primary endothelial cells, which play roles in latency establishment. J. Virol..

[204] Tripathi K., Mani C., Barnett R., Nalluri S., Bachaboina L., Rocconi R.P., Athar M., Owen L.B., Palle K. (2014). Gli1 protein regulates the S-phase checkpoint in tumor cells via bid protein, and its inhibition sensitizes to DNA topoisomerase 1 inhibitors. J. Biol. Chem..

[205] Abraham R.T. (2001). Cell cycle checkpoint signaling through the ATM and ATR kinases. Genes Dev..

[206] Mazumdar T., DeVecchio J., Agyeman A., Shi T., Houghton J.A. (2011). Blocking hedgehog survival signaling at the level of the GLI genes induces DNA damage and extensive cell death in human colon carcinoma cells. Cancer Res..

[207] Pak E., Segal R.A. (2016). Hedgehog signal transduction: Key players, oncogenic drivers, and cancer therapy. Dev. Cell.

[208] Balakrishnan L., Milavetz B. (2017). Epigenetic regulation of viral biological processes. Viruses.

[209] Qu D., Sun W.-W., Li L., Ma L., Sun L., Jin X., Li T., Hou W., Wang J.-H. (2019). Long noncoding RNA MALAT1 releases epigenetic silencing of HIV-1 replication by displacing the polycomb repressive complex 2 from binding to the LTR promoter. Nucleic Acids Res..

[210] Hung S.Y., Lin H.H., Yeh K.T., Chang J.G. (2014). Histone-modifying genes as biomarkers in hepatocellular carcinoma. Int. J. Clin. Exp. Pathol..

[211] Hu J.J., Song W., Zhang S.D., Shen X.-H., Qiu X.-M., Wu H.-Z., Gong P.-H., Lu S., Zhao Z.-J., He M.-L. (2016). HBx-upregulated lncRNA UCA1 promotes cell growth and tumorigenesis by recruiting EZH2 and repressing p27Kip1/CDK2 signaling. Sci. Rep..

[212] Tsang D.P., Wu W.K., Kang W., Lee Y.Y., Wu F., Yu Z., Xiong L., Chan K.W.A., Tong J., Yang W. (2016). Yin Yang 1-mediated epigenetic silencing of tumour-suppressive microRNAs activates nuclear factor-kappaB in hepatocellular carcinoma. J. Pathol..

[213] Schaeffner M., Mrozek-Gorska P., Buschle A., Woellmer A., Tagawa T., Cernilogar F.M., Schotta G., Krietenstein N., Lieleg C., Korber P. (2019). BZLF1 interacts with chromatin remodelers promoting escape from latent infections with EBV. Life Sci. Alliance.

[214] Kim S.H., Yang W.I., Min Y.H., Ko Y.H., Yoon S.O. (2015). The role of the polycomb repressive complex pathway in T and NK cell lymphoma: Biological and prognostic implications. Tumor Biol..

[215] Toth Z., Papp B., Brulois K., Choi Y.J., Gao S.-J., Jung J.U. (2016). LANA-mediated recruitment of host polycomb repressive complexes onto the KSHV genome during de novo infection. PLoS Pathog..

[216] He M., Zhang W., Bakken T., Schutten M., Toth Z., Jung J.U., Gill P., Cannon M., Gao S.J. (2012). Cancer angiogenesis induced by kaposi sarcoma–associated herpesvirus is mediated by EZH2. Cancer Res..

[217] Lan X., Wen H., Cheng K., Plagov A., Shoshtari S.S.M., Malhotra A., Singhal P.C. (2017). Hedgehog pathway plays a vital role in HIV-induced epithelial-mesenchymal transition of podocyte. Exp. Cell Res..

[218] Pereira T.A., Witek R.P., Syn W.K., Choi S.S., Bradrick S., Karaca G.F., Agboola K.M., Jung Y., Omenetti A., Moylan C.A. (2010). Viral factors induce Hedgehog pathway activation in humans with viral hepatitis, cirrhosis, and hepatocellular carcinoma. Lab. Investig..

[219] Granato M., Zompetta C., Vescarelli E., Rizzello C., Cardi A., Valia S., Antonelli G., Marchese C., Torrisi M.R., Faggioni A. (2016). HCV derived from sera of HCV-infected patients induces pro-fibrotic effects in human primary fibroblasts by activating GLI2. Sci. Rep..

[220] Kim H.Y., Cho H.K., Hong S.P., Cheong J. (2011). Hepatitis B virus X protein stimulates the Hedgehog-Gli activation through protein stabilization and nuclear localization of Gli1 in liver cancer cells. Cancer Lett..

[221] Arzumanyan A., Sambandam V., Clayton M.M., Choi S.S., Xie G., Diehl A.M., Yu D.Y., Feitelson M.A. (2012). Hedgehog signaling blockade delays hepatocarcinogenesis induced by hepatitis B virus X protein. Cancer Res..

[222] Choi S.S., Bradrick S., Qiang G., Mostafavi A., Chaturvedi G., Weinman S.A., Diehl A.M., Jhaveri R. (2011). Up-regulation of Hedgehog pathway is associated with cellular permissiveness for hepatitis C virus replication. Hepatology.

[223] Cooper C.L., Hardy R.R., Reth M., Desiderio S. (2012). Non-cell-autonomous hedgehog signaling promotes murine B lymphopoiesis from hematopoietic progenitors. Blood.

[224] Siggins S.L., Nguyen N.Y., McCormack M.P., Vasudevan S., Villani R., Jane S.M., Wainwright J.B., Curtis J.D. (2009). The Hedgehog receptor Patched1 regulates myeloid and lymphoid progenitors by distinct cell-extrinsic mechanisms. Blood.

[225] Trowbridge J.J., Scott M.P., Bhatia M. (2006). Hedgehog modulates cell cycle regulators in stem cells to control hematopoietic regeneration. Proc. Natl. Acad. Sci. USA.

[226] Gering M., Patient R. (2005). Hedgehog signaling is required for adult blood stem cell formation in zebrafish embryos. Dev. Cell.

[227] Rowe M., Glaunsinger B., Van Leeuwen D., Zuo J., Sweetman D., Ganem D., Middeldorp J., Wiertz E., Ressing M.E. (2007). Host shutoff during productive Epstein-Barr virus infection is mediated by BGLF5 and may contribute to immune evasion. Proc. Natl. Acad. Sci. USA.

[228] Port R.J., Pinheiro-Maia S., Hu C., Arrand J.R., Wei W., Young L.S., Dawson C.W. (2013). Epstein-Barr virus induction of the Hedgehog signalling pathway imposes a stem cell phenotype on human epithelial cells. J. Pathol..

[229] Deb Pal A., Banerjee S. (2015). Epstein-Barr virus latent membrane protein 2A mediated activation of Sonic Hedgehog pathway induces HLA class Ia downregulation in gastric cancer cells. Virology.

[230] Furler R.L., Uittenbogaart C.H. (2012). GLI2 regulates TGF-beta1 in human CD4+ T cells: Implications in cancer and HIV pathogenesis. PLoS ONE.

[231] Yoshida T., Hamano A., Ueda A., Takeuchi H., Yamaoka S. (2017). Human SMOOTHENED inhibits human immunodeficiency virus type 1 infection. Biochem. Biophys. Res. Commun..

[232] Syn W.K., Choi S.S., Liaskou E., Karaca G.F., Agboola K.M., Oo Y.H., Mi Z., Pereira T.A., Zdanowicz M., Malladi P. (2010). Osteopontin is induced by hedgehog pathway activation and promotes fibrosis progression in nonalcoholic steatohepatitis. Hepatology.

[233] Navas M.C., Glaser S., Dhruv H., Celinski S., Alpini G., Meng F. (2019). Hepatitis C virus infection and cholangiocarcinoma: An insight into epidemiologic evidences and hypothetical mechanisms of oncogenesis. Am. J. Pathol..

[234] Kuromi T., Matsushita M., Iwasaki T., Nonaka D., Kuwamoto S., Nagata K., Kato M., Akizuki G., Kitamura Y., Hayashi K. (2017). Association of expression of the hedgehog signal with Merkel cell polyomavirus infection and prognosis of Merkel cell carcinoma. Hum. Pathol..

[235] Rojo-León V., García C., Valencia C., Méndez M.A., Wood C.D., Covarrubias L. (2019). The E6/E7 oncogenes of human papilloma virus and estradiol regulate hedgehog signaling activity in a murine model of cervical cancer. Exp. Cell Res..

[236] Enzenhofer E., Parzefall T., Haymerle G., Schneider S., Kadletz L., Heiduschka G., Pammer J., Oberndorfer F., Wrba F., Loader B. (2016). Impact of sonic hedgehog pathway expression on outcome in HPV negative head and neck carcinoma patients after surgery and adjuvant radiotherapy. PLoS ONE.

[237] Solecki D.J., Gromeier M., Mueller S., Bernhardt G., Wimmer E. (2002). Expression of the human poliovirus receptor/CD155 gene is activated by sonic hedgehog. J. Biol. Chem..

[238] Kim S.H., Choe J.-Y., Jeon Y., Huh J., Jung H.R., Choi Y.-D., Kim H.-J., Cha H.J., Park W.S., Kim J.E. (2013). Frequent expression of follicular dendritic cell markers in Hodgkin lymphoma and anaplastic large cell lymphoma. J. Clin. Pathol..

[239] Asha K., Balfe N., Sharma-Walia N. (2020). Concurrent control of the kaposi’s sarcoma-associated herpes-virus life cycle through chromatin modulation and host hedgehog signaling: A new prospect for the therapeutic potential of lipoxin A4. J. Virol..

[240] Tanimura A., Dan S., Yoshida M. (1998). Cloning of novel isoforms of the human Gli2 oncogene and their activities to enhance tax-dependent transcription of the human T-cell leukemia virus type 1 genome. J. Virol..

[241] Shin Y., Lim H., Choi B.S., Kim K.C., Kang C., Bae Y.S., Yoon C.-H. (2016). Highly activated p53 contributes to selectively increased apoptosis of latently HIV-1 infected cells upon treatment of anticancer drugs. Virol. J..

[242] Mitchell J.K., Midkiff B.R., Israelow B., Evans M.J., Lanford R.E., Walker C.M., Lemon S.M., McGivern D.R. (2017). Hepatitis C virus indirectly disrupts DNA damage-induced p53 responses by activating protein kinase, R. mBio.

[243] Tornesello M.L., Annunziata C., Tornesello A.L., Buonaguro F.M., Buonaguro L. (2018). Human oncoviruses and p53 tumor suppressor pathway deregulation at the origin of human cancers. Cancers.

[244] Liu Y., Qi X., Zeng Z., Wang L., Wang J., Zhang T., Xu Q., Shen C., Zhou G., Yang S. (2017). CRISPR/Cas9-mediated p53 and Pten dual mutation accelerates hepatocarcinogenesis in adult hepatitis B virus transgenic mice. Sci. Rep..

[245] Ganguly S., Kuravi S., Alleboina S., Mudduluru G., Jensen R.A., McGuirk J.P., Balusu R. (2019). Targeted therapy for EBV-associated B-cell neoplasms. Mol. Cancer Res..

[246] Sarek G., Ma L., Enback J., Jarviluoma A., Moreau P., Haas J., Laiho M., Ojala P.M. (2013). Kaposi’s sarcoma herpesvirus lytic replication compromises apoptotic response to p53 reactivation in virus-induced lymphomas. Oncogene.

[247] Wang Z., Shang H., Jiang Y. (2017). Chemokines and chemokine receptors: Accomplices for human immunodeficiency virus infection and latency. Front. Immunol..

[248] Chen F., Zhang J., Wen B., Luo S., Lin Y., Ou W., Guo F., Tang P., Liu W., Qu X. (2016). HBV/HCV dual infection impacts viral load, antibody response, and cytokine expression differently from HBV or HCV single infection. Sci. Rep..

[249] Elia G., Fallahi P. (2017). Hepatocellular carcinoma and CXCR3 chemokines: A narrative review. La Clin. Ter..

[250] Stine J.T., Wood C., Hill M., Epp A., Raport C.J., Schweickart V.L., Endo Y., Sasaki T., Simmons G., Boshoff C. (2000). KSHV-encoded CC chemokine vMIP-III is a CCR4 agonist, stimulates angiogenesis, and selectively chemoattracts TH2 cells. Blood.

[251] Boshoff C., Endo Y., Collins P.D., Takeuchi Y., Reeves J.D., Schweickart V.L., Siani M.A., Sasaki T., Williams T.J., Gray P.W. (1997). Angiogenic and HIV-inhibitory functions of KSHV-encoded chemokines. Science.

[252] Del Corno M., Donninelli G., Varano B., Da Sacco L., Masotti A., Gessani S. (2014). HIV-1 gp120 activates the STAT3/interleukin-6 axis in primary human monocyte-derived dendritic cells. J. Virol..

[253] Sternberg C., Gruber W., Eberl M., Tesanovic S., Stadler M., Elmer D.P., Schlederer M., Grund S., Roos S., Wolff F. (2018). Synergistic cross-talk of hedgehog and interleukin-6 signaling drives growth of basal cell carcinoma. Int. J. Cancer.

[254] An J., Lichtenstein A.K., Brent G., Rettig M.B. (2002). The Kaposi sarcoma-associated herpesvirus (KSHV) induces cellular interleukin 6 expression: Role of the KSHV latency-associated nuclear antigen and the AP1 response element. Blood.

[255] Paquette S.G., Banner D., Zhao Z., Fang Y., Huang S.S.H., Leόn A.J., Ng D.C.K., Almansa R., Martin-Loeches I., Ramirez P. (2012). Interleukin-6 is a potential biomarker for severe pandemic H1N1 influenza an infection. PLoS ONE.

[256] Percopo C.M., Ma M., Brenner T., Krumholz J.O., Break T.J., Laky K., Rosenberg H.F. (2019). Critical adverse impact of IL-6 in acute pneumovirus infection. J. Immunol..

[257] Xia C., Liu Y., Chen Z., Zheng M. (2015). Involvement of interleukin 6 in hepatitis B viral infection. Cell. Physiol. Biochem..

[258] Yokoi T., Miyawaki T., Yachie A., Kato K., Kasahara Y., Taniguchi N. (1990). Epstein-Barr virus-immortalized B cells produce IL-6 as an autocrine growth factor. Immunology.

[259] Seto M., Ohta M., Asaoka Y., Ikenoue T., Tada M., Miyabayashi K., Mohri D., Tanaka Y., Ijichi H., Tateishi K. (2009). Regulation of the hedgehog signaling by the mitogen-activated protein kinase cascade in gastric cancer. Mol. Carcinog..

[260] Kasperczyk H., Baumann B., Debatin K.M., Fulda S. (2009). Characterization of sonic hedgehog as a novel NF-kappaB target gene that promotes NF-kappaB-mediated apoptosis resistance and tumor growth in vivo. FASEB J..

[261] Polizio A.H., Chinchilla P., Chen X., Kim S., Manning D.R., Riobo N.A. (2011). Heterotrimeric Gi proteins link hedgehog signaling to activation of rho small GTPases to promote fibroblast migration. J. Biol. Chem..

[262] Kasai K., Takahashi M., Osumi N., Sinnarajah S., Takeo T., Ikeda H., Kehrl J.H., Itoh G., Arnheiter H. (2004). The G12 family of heterotrimeric G proteins and Rho GTPase mediate Sonic hedgehog signalling. Genes Cells.

[263] Toschi E., Bacigalupo I., Strippoli R., Chiozzini C., Cereseto A., Falchi M., Nappi F., Sgadari C., Barillari G., Paolini R. (2006). HIV-1 tat regulates endothelial cell cycle progression via activation of the Ras/ERK MAPK signaling pathway. Mol. Biol. Cell.

[264] Liu Z., Tian Y., Machida K., Lai M.M.C., Luo G., Foung S.K.H., Ou J.H.J. (2012). Transient Activation of the PI3K-AKT Pathway by Hepatitis C Virus to Enhance Viral Entry. J. Biol. Chem..

[265] Chen Y., Bai X., Zhang Q., Wen L., Su W., Fu Q., Sun X., Lou Y., Yang J., Zhang J. (2016). The hepatitis B virus X protein promotes pancreatic cancer through modulation of the PI3K/AKT signaling pathway. Cancer Lett..

[266] Roberts M., Cooper N.R. (1998). Activation of a ras–MAPK-dependent pathway by epstein–barr virus latent membrane protein 1 is essential for cellular transformation. Virology.

[267] Paul A.G., Chandran B., Sharma-Walia N. (2013). Cyclooxygenase-2-prostaglandin E2-eicosanoid receptor inflammatory axis: A key player in Kaposi’s sarcoma-associated herpes virus associated malignancies. Transl. Res..

[268] Sharma-Walia N., Krishnan H.H., Naranatt P.P., Zeng L., Smith M.S., Chandran B. (2005). ERK1/2 and MEK1/2 induced by Kaposi’s sarcoma-associated herpesvirus (human herpesvirus 8) early during infection of target cells are essential for expression of viral genes and for establishment of infection. J. Virol..

[269] Chandran B. (2010). Early events in Kaposi’s sarcoma-associated herpesvirus infection of target cells. J. Virol..

[270] Campbell G.R., Bruckman R.S., Herns S.D., Joshi S., Durden D.L., Spector S.A. (2018). Induction of autophagy by PI3K/MTOR and PI3K/MTOR/BRD4 inhibitors suppresses HIV-1 replication. J. Biol. Chem..

[271] Golob-Schwarzl N., Krassnig S., Toeglhofer A.M., Park Y.N., Gogg-Kamerer M., Vierlinger K., Schröder F., Rhee H., Schicho R., Fickert P. (2017). New liver cancer biomarkers: PI3K/AKT/mTOR pathway members and eukaryotic translation initiation factors. Eur. J. Cancer.

[272] Stöhr S., Costa R., Sandmann L., Westhaus S., Pfaender S., Anggakusuma, Dazert E., Meuleman P., Vondran F.W.R., Manns M.P. (2016). Host cell mTORC1 is required for HCV RNA replication. Gut.

[273] Liu X., Hu X., Kuang Y., Yan P., Li L., Li C., Tao Q., Cai X. (2017). BCLB, methylated in hepatocellular carcinoma, is a starvation stress sensor that induces apoptosis and autophagy through the AMPK-mTOR signaling cascade. Cancer Lett..

[274] Wu S.-X., Chen W.-N., Jing Z.T., Liu W., Lin X.J., Lin X. (2018). Hepatitis B spliced protein (HBSP) suppresses fas-mediated hepatocyte apoptosis via activation of PI3K/Akt signaling. J. Virol..

[275] Xiang K., Wang B. (2018). Role of the PI3K‑AKT‑mTOR pathway in hepatitis B virus infection and replication. Mol. Med. Rep..

[276] Chang H.H., Ganem D. (2013). A unique herpesviral transcriptional program in KSHV-infected lymphatic endothelial cells leads to mTORC1 activation and rapamycin sensitivity. Cell Host Microbe.

[277] Bhatt A.P., Damania B. (2013). AKTivation of PI3K/AKT/mTOR signaling pathway by KSHV. Front. Immunol..

[278] Priya Aravinth S., Rajendran S., Li Y., Wu M., Yi Wong A.H., Schwarz H. (2019). Epstein-Barr virus-encoded LMP1 induces ectopic CD137 expression on Hodgkin and Reed-Sternberg cells via the PI3K-AKT-mTOR pathway. Leuk Lymphoma..

[279] Cohen J.I. (2018). Herpesviruses in the activated phosphatidylinositol-3-kinase-delta syndrome. Front Immunol..

[280] Zhong Y., Hennig B., Toborek M. (2009). Intact lipid rafts regulate HIV-1 tat protein-induced activation of the rho signaling and upregulation of P-glycoprotein in brain endothelial cells. Br. J. Pharmacol..

[281] Budzko L., Marcinkowska-Swojak M., Jackowiak P., Kozlowski P., Figlerowicz M. (2016). Copy number variation of genes involved in the hepatitis C virus-human interactome. Sci. Rep..

[282] Ma W., Wong C.C., Tung E.K., Wong C.M., Ng I.O. (2013). RhoE is frequently down-regulated in hepatocellular carcinoma (HCC) and suppresses HCC invasion through antagonizing the Rho/Rho-kinase/myosin phosphatase target pathway. Hepatology.

[283] Tugizov S.M., Herrera R., Palefsky J.M. (2013). Epstein-barr virus transcytosis through polarized oral epithelial cells. J. Virol..

[284] Lawson B., Franklinos L.H.V., Fernandez J.R.R., Wend-Hansen C., Nair S., MacGregor S.K., John S.K., Pizzi R., Núñez A., Ashton P.M. (2018). Salmonella Enteritidis ST183: Emerging and endemic biotypes affecting western European hedgehogs (Erinaceus europaeus) and people in Great Britain. Sci. Rep..

[285] Shi X., Wei S., Simms K.J., Cumpston D.N., Ewing T.J., Zhang P. (2018). Sonic hedgehog signaling regulates hematopoietic stem/progenitor cell activation during the granulopoietic response to systemic bacterial infection. Front. Immunol..

[286] Schumacher M.A., Feng R., Aihara E., Engevik A.C., Montrose M.H., Ottemann K.M., Zavros Y. (2015). Helicobacter pylori-induced Sonic Hedgehog expression is regulated by NFkappaB pathway activation: The use of a novel in vitro model to study epithelial response to infection. Helicobacter.

[287] Shiotani A., Iishi H., Uedo N., Ishihara R., Ishiguro S., Tatsuta M., Nakae Y., Kumamoto M., Hinoi T., Merchant J.L. (2006). Helicobacter pylori-induced atrophic gastritis progressing to gastric cancer exhibits sonic hedgehog loss and aberrant CDX2 expression. Aliment. Pharmacol. Ther..

[288] Feng R., Xiao C., Zavros Y. (2012). The role of Sonic Hedgehog as a regulator of gastric function and differentiation. Vitam. Horm..

[289] Scales S.J., de Sauvage F.J. (2009). Mechanisms of Hedgehog pathway activation in cancer and implications for therapy. Trends Pharmacol. Sci..

[290] Sharpe H.J., Wang W., Hannoush R.N., De Sauvage F.J. (2015). Regulation of the oncoprotein Smoothened by small molecules. Nat. Chem. Biol..

[291] Bar E.E., Chaudhry A., Lin A., Fan X., Schreck K., Matsui W., Piccirillo S., Vescovi A.L., DiMeco F., Olivi A. (2007). Cyclopamine-mediated hedgehog pathway inhibition depletes stem-like cancer cells in glioblastoma. Stem Cells.

[292] Chen J.K., Taipale J., Cooper M.K., Beachy P.A. (2002). Inhibition of Hedgehog signaling by direct binding of cyclopamine to Smoothened. Genes Dev..

[293] Ng J.M., Curran T. (2011). The Hedgehog’s tale: Developing strategies for targeting cancer. Nat. Rev. Cancer.

[294] Sverrisson E.F., Zens M.S., Fei D.L., Andrews A., Schned A., Robbins D., Kelsey K.T., Li H., DiRenzo J., Karagas M.R. (2014). Clinicopathological correlates of Gli1 expression in a population-based cohort of patients with newly diagnosed bladder cancer. Urol. Oncol..

[295] Mikami Y., Fujii S., Nagata K., Wada H., Hasegawa K., Abe M., Yoshimoto R.U., Kawano S., Nakamura S., Kiyoshima T. (2017). GLI-mediated Keratin 17 expression promotes tumor cell growth through the anti-apoptotic function in oral squamous cell carcinomas. J. Cancer Res. Clin. Oncol..

[296] Johnson R.W., Nguyen M.P., Padalecki S.S., Grubbs B.G., Merkel A.R., Oyajobi B.O., Matrisian L.M., Mundy G.R., Sterling J.A. (2011). TGF-β promotion of Gli2-induced expression of parathyroid hormone-related protein, an important osteolytic factor in bone metastasis, is independent of canonical Hedgehog signaling. Cancer Res..

[297] Song S., Jiang J., Zhao L., Wang Q., Lu W., Zheng C., Zhang J., Ma H., Tian S., Zheng J. (2019). Structural optimization on a virtual screening hit of smoothened receptor. Eur. J. Med. Chem..

[298] Dheeraj A., Rigby C.M., O’Bryant C.L., Agarwal C., Singh R.P., Deep G., Agarwal R. (2017). Silibinin treatment inhibits the growth of hedgehog inhibitor-resistant basal cell carcinoma cells via targeting EGFR-MAPK-Akt and hedgehog signaling. Photochem. Photobiol..

[299] Kremer M.S.L., Schultz-Fademrecht C., Baumann M., Habenberger P., Choidas A., Klebl B., Kordes S., Schöler H.R., Sterneckert J., Ziegler S. (2017). Discovery of a novel inhibitor of the hedgehog signaling pathway through cell-based compound discovery and target prediction. Angew. Chem. Int. Ed..

[300] Martínez C., Cornejo V.H., Lois P., Ellis T., Solis N.P., Wainwright B., Palma V. (2013). Proliferation of murine midbrain neural stem cells depends upon an endogenous sonic hedgehog (Shh) Source. PLoS ONE.

[301] Booker B.E., Steg A.D., Kovac S., Landen C.N., Amm H.M. (2020). The use of hedgehog antagonists in cancer therapy: A comparison of clinical outcomes and gene expression analyses. Cancer Biol. Ther..

[302] He M., Fu Y., Yan Y., Xiao Q., Wu H., Yao W., Zhao H., Zhao L., Jiang Q., Yu Z. (2015). The Hedgehog signalling pathway mediates drug response of MCF-7 mammosphere cells in breast cancer patients. Clin. Sci..

[303] Yang D., Cao F., Ye X., Zhao H., Liu X., Li Y., Shi C., Wang H., Zhou J. (2013). Arsenic trioxide inhibits the hedgehog pathway which is aberrantly activated in acute promyelocytic leukemia. Acta Haematol..

[304] Ma Y., Yu W., Shrivastava A., Alemi F., Lankachandra K., Srivastava R.K., Shankar S. (2017). Sanguinarine inhibits pancreatic cancer stem cell characteristics by inducing oxidative stress and suppressing sonic hedgehog-Gli-Nanog pathway. Carcinogenesis.

[305] Shord S.S., Casey D., Zhao H., Demko S., Keegan P., Pazdur R. (2017). FDA approval summary: Sonidegib—Response. Clin. Cancer Res..

[306] Gyawali B., Ando Y. (2017). FDA approval summary: Sonidegib—Letter. Clin. Cancer Res..

[307] Ming J., Sun B., Li Z., Lin L., Meng X., Han B., Wang R., Wu P., Li J., Cai J. (2017). Aspirin inhibits the SHH/GLI1 signaling pathway and sensitizes malignant glioma cells to temozolomide therapy. Aging.

